# Cardiac Actions of a Small Molecule Inhibitor Targeting GATA4–NKX2-5 Interaction

**DOI:** 10.1038/s41598-018-22830-8

**Published:** 2018-03-15

**Authors:** Sini M. Kinnunen, Marja Tölli, Mika J. Välimäki, Erhe Gao, Zoltan Szabo, Jaana Rysä, Mónica P. A. Ferreira, Pauli Ohukainen, Raisa Serpi, Alexandra Correia, Ermei Mäkilä, Jarno Salonen, Jouni Hirvonen, Hélder A. Santos, Heikki Ruskoaho

**Affiliations:** 1 Drug Research Program, Division of Pharmacology and Pharmacotherapy, University of Helsinki, Helsinki, Finland; 2 Department of Pharmacology and Toxicology, Institute of Biomedicine, University of Oulu, Oulu, Finland; 3 Lewis Katz School of Medicine at Temple University, Philadelphia, Pennsylvania United States of America; 4 School of Pharmacy, Faculty of Health Sciences, University of Eastern Finland, Kuopio, Finland; 5 Drug Research Program, Division of Pharmaceutical Chemistry and Technology, Faculty of Pharmacy, University of Helsinki, Helsinki, Finland; 6 Computational Medicine, Faculty of Medicine, University of Oulu and Biocenter Oulu, Oulu, Finland; 7 Laboratory of Industrial Physics, Department of Physics and Astronomy, University of Turku, Turku, Finland; 8 Helsinki Institute of Life Sciences (HiLIFE), University of Helsinki, Helsinki, Finland

## Abstract

Transcription factors are fundamental regulators of gene transcription, and many diseases, such as heart diseases, are associated with deregulation of transcriptional networks. In the adult heart, zinc-finger transcription factor GATA4 is a critical regulator of cardiac repair and remodelling. Previous studies also suggest that NKX2-5 plays function role as a cofactor of GATA4. We have recently reported the identification of small molecules that either inhibit or enhance the GATA4–NKX2-5 transcriptional synergy. Here, we examined the cardiac actions of a potent inhibitor (3i-1000) of GATA4–NKX2-5 interaction in experimental models of myocardial ischemic injury and pressure overload. In mice after myocardial infarction, 3i-1000 significantly improved left ventricular ejection fraction and fractional shortening, and attenuated myocardial structural changes. The compound also improved cardiac function in an experimental model of angiotensin II -mediated hypertension in rats. Furthermore, the up-regulation of cardiac gene expression induced by myocardial infarction and ischemia reduced with treatment of 3i-1000 or when micro- and nanoparticles loaded with 3i-1000 were injected intramyocardially or intravenously, respectively. The compound inhibited stretch- and phenylephrine-induced hypertrophic response in neonatal rat cardiomyocytes. These results indicate significant potential for small molecules targeting GATA4–NKX2-5 interaction to promote myocardial repair after myocardial infarction and other cardiac injuries.

## Introduction

Heart disease is one of the most serious challenges for modern medicine. Heart failure affects more than 37 million people globally and its prevalence is rapidly growing^1^. The total medical costs in the USA heart failure patients were $20.9 billion in 2012 and are predicted to rise to $53.1 billion by 2030^1^. The key pathophysiological process that ultimately leads to heart failure is myocardial remodelling^2^. Common causes include disorders that chronically increase cardiac workload, such as loss of myocytes due to myocardial infarction (MI) or pressure overload due to hypertension. Current therapy of myocardial remodelling is based on targeting mechanical and humoral mechanisms (*e.g*., by angiotensin-converting-enzyme inhibitors, angiotensin receptor blockers, beta-blockers, diuretics and mineralocorticoid receptor antagonists)^3^. Despite optimal treatment with the existing drugs, the rates of morbidity and mortality are still high in patients with heart failure^1^, and novel therapeutic strategies are necessary to prevent and reverse myocardial remodelling.

Key molecules that have emerged as highly promising targets for therapeutic manipulation include transcription factors (TFs). TFs are adaptor molecules that detect regulatory sequences in the DNA and target the assembly of protein complexes that control gene expression^4^. In the heart, the master TFs GATA4 and NKX2-5 are required for cardiogenesis^5^. In the adult heart, GATA4 is critical regulator of cardiac repair and regeneration. Heart-specific deletion of GATA4 reduces the hypertrophic response to pressure overload and rapidly leads to impaired cardiac function^6–8^. GATA4 heterozygous mice are also more susceptible to heart failure and show enhanced cardiac injury in response to doxorubicin administration^9,10^. Moreover, reversal of reduced GATA4 activity by adenovirus-mediated gene transfer has reported to prevent adverse post-infarction remodelling through myocardial angiogenesis, anti-apoptosis, and stem cell recruitment^11^. Similarly to GATA4^12–14^, also the expression of NKX2-5 is up-regulated by hypertrophic stimuli^4^. GATA4 and NKX2-5 interact physically and act synergistically as key transcriptional controllers of numerous cardiac genes, including atrial natriuretic peptide (ANP, also known as NPPA)^15–18^. Importantly, previous mutational studies have shown that the GATA consensus sites in combination with an NKX2-5 binding element are important for the mechanical stretch-induced B-type natriuretic peptide (BNP, also known as NPPB) gene activation and cardiomyocyte hypertrophy^19^. This finding implicates that the GATA4–NKX2-5 interaction is essential for the response of cardiomyocytes to external stimuli, such as a hemodynamic overload due to high blood pressure and myocardial infarction.

Recently, we have reported the identification of four small molecule families that either inhibit or enhance the GATA4–NKX2-5 transcriptional synergy^20^. Multiple research methods i.e. fragment based screening, reporter gene assay measuring transcriptional synergy, and pharmacophore search were utilized for small molecule screening, identification, and optimization. Here, we examined the actions of the most potent inhibitor (3i-1000) of GATA4–NKX2-5 interaction in *in vitro* and *in vivo* experimental models of ischemic injury and pressure overload and found cardioprotective actions. Our results implicate that modulators of protein–protein interactions of key transcription factors may present one of the next classes of innovative therapeutic targets.

## Results

### Inhibition of GATA4–NKX2-5 interaction by a small molecule 3i-1000

**Figure 1:**
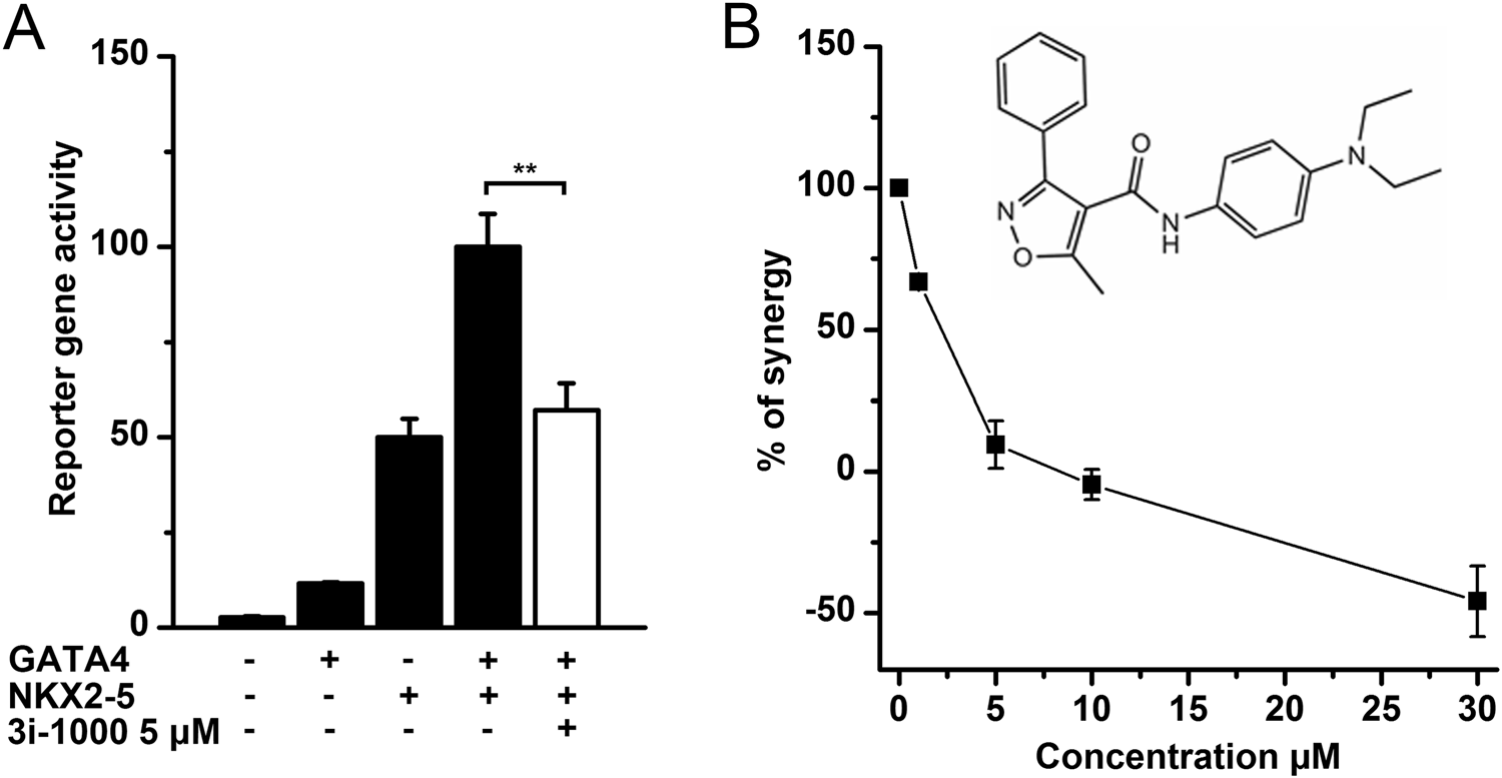
The effect of small molecule 3i-1000 on GATA–NKX2-5 interaction in cell-based reporter gene assay. (**A**) COS-1 cells were transfected with a reporter construct containing three high-activation binding sites for NKX2-5 together with protein expression vectors for GATA4 and NKX2-5. The cells were lysed, and the reporter gene activity was measured by a luminometer. The small molecule 3i-1000 inhibited GATA4–NKX2-5 transcriptional synergy in luciferase reporter assay at the concentration of 5 µM. The results are an average of three parallel samples ± SD. **p < 0.01 (independent samples Student’s *t*-test). (**B**) Dose-dependent inhibition of the GATA4–NKX2-5 transcriptional synergy with the small molecule 3i-1000. The molecular structure of the compound is also shown. The results are an average of two experiments with 4 or 8 replicates ± SEM.

The GATA4–NKX2-5 interaction was used as a target to design small molecules to disturb the protein–protein interactions of these highly conserved TFs. The homology model of the interaction was defined by mutating several amino acids on the surface of GATA4^21^. As a primary screening method, we used a luciferase reporter assay specifically prepared for the GATA4–NKX2-5 interaction^20,21^. In this assay, the luciferase gene is encoded by an artificial promoter containing three high affinity binding sites for NKX2-5 (p3xHA-luc), and together with GATA4, the transcription of the gene is activated synergistically (Fig. 1A). By combining experimental and computational methods, we were able to identify four compound families with either antagonistic or agonistic effect on GATA4–NKX2-5 induced synergistic activation^20^. The most potent compound (N-4-(diethylamino)phenyl)-5-methyl-3phenylisoxazole-4-carboxamide (compound 3, here nominated 3i-1000)^20^ inhibited GATA4–NKX2-5 transcriptional synergy dose-dependently (Fig. 1B).

### 3i-1000 inhibits cardiac gene expression induced by mechanical stretch and phenylephrine

**Figure 2:**
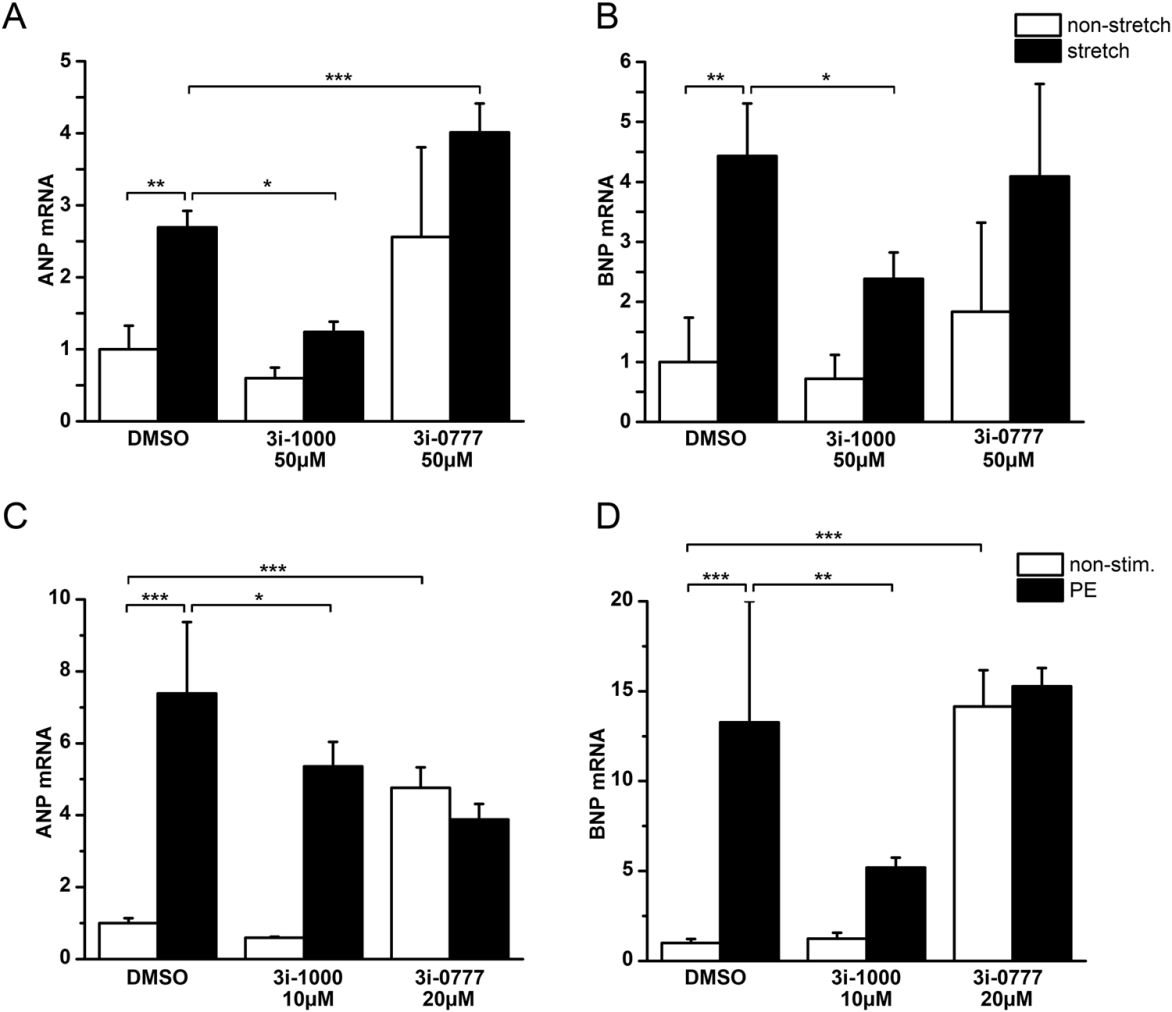
The effect of small molecules acting on GATA4–NKX2-5 transcriptional synergy on the hypertrophic process *in vitro* in neonatal cardiac myocytes. (**A,B**) The effects of 3i-1000 and 3i-0777 on stretch-induced increase in ANP (**A**) and (**B**) BNP mRNA levels. Cultured neonatal rat cardiomyocytes were stretched cyclically up to 24 h. The compounds were added 1 h prior stretching to the cells. (**C,D**) Effects of the compounds on phenylephrine (PE) induced increase in ANP (**C**) and (**D**) BNP gene expression. Cells were treated for 24 h with PE and the compounds were added 1 h prior to PE. mRNA levels were measured by RT-PCR and normalised to housekeeping gene 18 S quantified from the same samples. The results are averages ± SD, *n* = 3. *p < 0.05, **p < 0.01, ***p < 0.001 (one-way ANOVA followed by a least significant difference post hoc test).

Mechanical stretch induces hypertrophic growth of the cardiac myocytes, as reflected by the increase of cardiomyocyte cell size and activation of the foetal genes, such as ANP and BNP^19,20^. We have previously reported that the tandem GATA consensus sites of the proximal promoter in combination with the NKX2-5 binding element are critical for stretch-activated BNP transcription and sarcomere reorganisation^19^. Therefore, we first examined the effect of 3i-1000 on natriuretic peptide gene expression by employing an *in vitro* mechanical stretch model of cultured neonatal rat cardiomyocytes^19^. Interestingly, 3i-1000 decreased mechanical stretch-activated ANP (Fig. 2A) and BNP (Fig. 2B) gene expression at micromolar concentrations without significantly influencing the baseline ANP and BNP mRNA levels. We also tested the effect of 3i-1000 on the induction of expression of ANP and BNP genes by the hypertrophic agonist phenylephrine (PE) *in vitro* on neonatal rat cardiomyocytes. As shown in Fig. 2C and D, PE alone markedly increased ANP and BNP mRNA levels, and these increases in natriuretic peptide gene expression were significantly decreased with 3i-1000. Taken together, these results indicate that 3i-1000 is able to inhibit hypertrophic process in cardiomyocytes *in vitro*.

During the small molecule screening, we identified also a compound (compound 7, here nominated 3i-0777)^20^ that strongly augmented the GATA4–NKX2-5 synergy in the reporter gene assay. In contrast to the compound 3i-1000, the activator 3i-0777 significantly enhanced the mechanical stretch-stimulated increase of the ANP mRNA levels (Fig. 2A). Moreover, small molecule activator of GATA4–NKX2-5 transcriptional synergy increased significantly also the baseline ANP and BNP mRNA levels at 20 µM concentration (Fig. 2C–D).

### The effect of 3i-1000 on GATA4 phosphorylation

**Figure 3:**
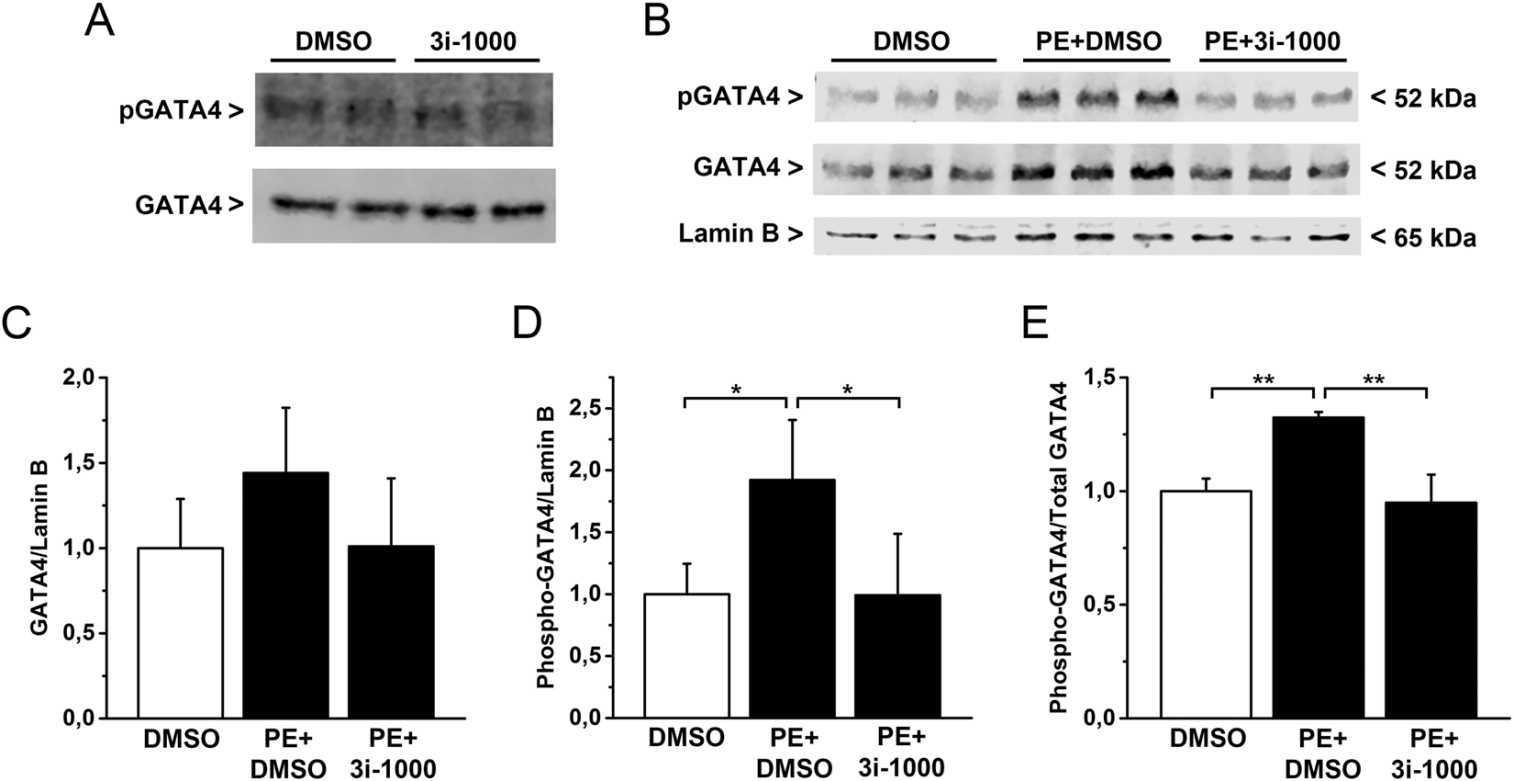
The effect of small molecule 3i-1000 on GATA4 protein levels and phosphorylation *in vitro* in neonatal rat cardiomyocytes. (**A**) Compound 3i-1000 at concentration of 50 µM had no influence on baseline levels of nuclear GATA4 or Ser-105 phosphorylation of GATA4 (pGATA4) protein. (**B–E**) Compound 3i-1000 (50 µM) inhibited the elevation of GATA4 and phospho-GATA4 protein levels produced with PE. The experiment was repeated three times, and the results presented here are an average of three parallel samples ± SD. The original whole blot images are presented in Supplementary Figure S1. *p < 0.05, **p < 0.01 (one-way ANOVA followed by a least significant difference post hoc test).

In addition to interactions with a number of TFs and co-factors, the transcriptional activity of GATA4 is regulated through posttranslational modifications^5^. Treatment of neonatal rat cardiomyocytes with hypertrophic agonists, such as PE and endothelin-1, increases the GATA4 phosphorylation via mitogen activated protein kinase (MAPK) pathways, leading to an increase in GATA4 activity^5^. To further characterize the effects of 3i-1000, we investigated its actions on GATA4 phosphorylation induced by PE using Western blotting. The original images of the whole blots are presented in Supplementary Fig. S1. As shown in Fig. 3A, the compound had no influence on the baseline GATA4 proteins levels in neonatal rat cardiomyocytes, whereas the PE-induced elevation in GATA4 Ser-105 phosphorylation was significantly inhibited by 3i-1000 (Fig. 3B–E). This reduction of the increased phosphorylated GATA4 protein in response to PE is in agreement with the observations that 3i-1000 inhibited stretch- and PE-induced natriuretic peptide gene expression in cardiomyocytes.

### Cardiac actions of 3i-1000 in experimental models of myocardial infarction in mice and rats

To investigate the effects of the small molecule inhibitor of GATA4–NKX2-5 interaction *in vivo*, we first tested the pharmacokinetics and metabolic profile of 3i-1000 in normal rats by injecting a single dose i.p. (10 mg/kg). The blood samples were collected at 0.5 h, 2 h and 6 h after injection. The concentration of 3i-1000 was highest at 0.5 h and decreased to about half within 6 h, indicating rapid metabolism of the 3i-1000 *in vivo* in rats (Supplementary Fig. S2). Overall, the compound was well tolerated and no signs of toxicity was observed, and when we analysed the blood samples after two weeks administration (30 mg/kg/day i.p.), only minor changes in blood cells were noted (data not shown).

**Figure 4:**
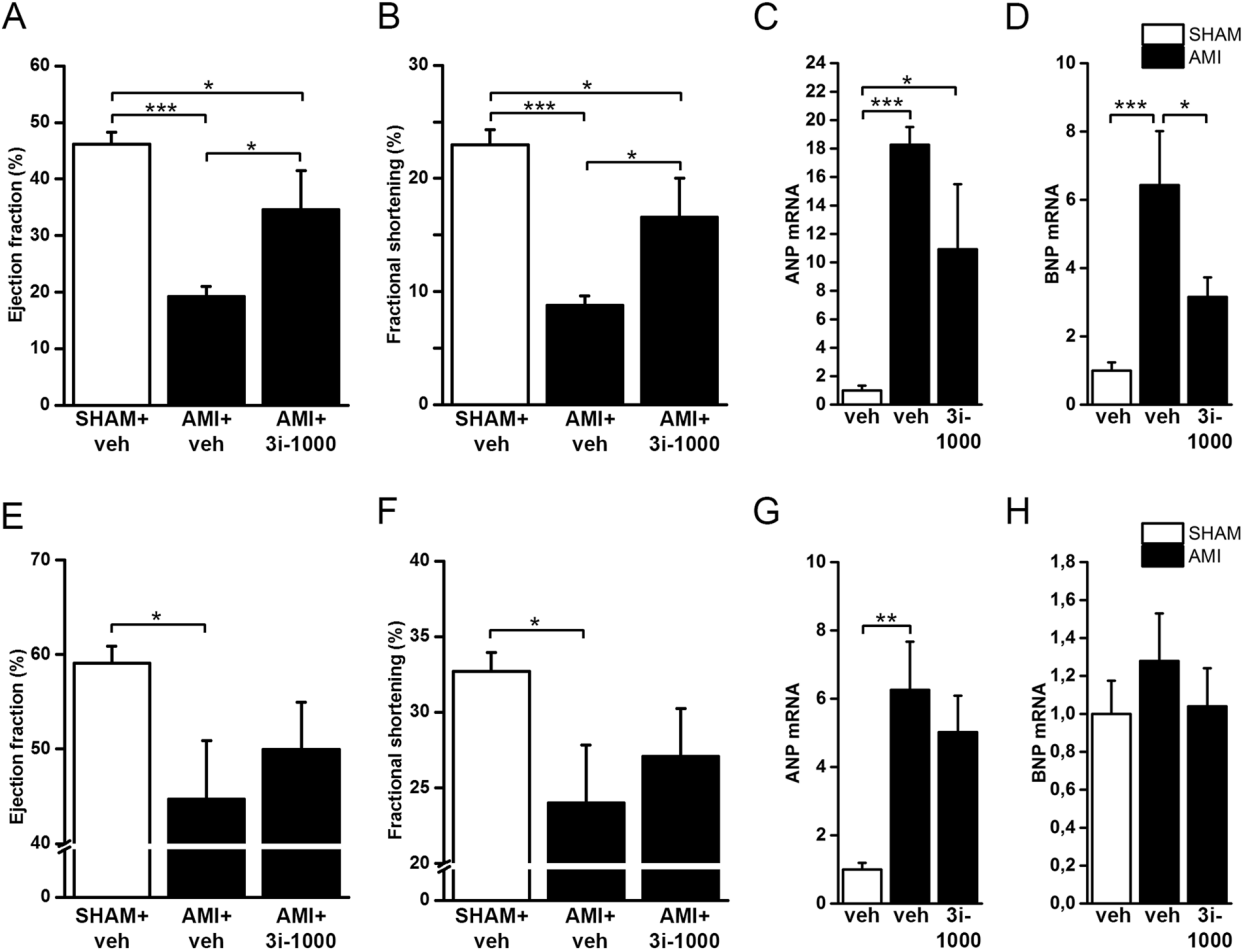
(**A–D**) The echocardiographic parameters and mRNA levels in the left ventricular tissue of mice that underwent acute myocardial infarction (AMI) or sham-operation (SHAM) and were treated either with vehicle (veh) or compound 3i-1000 (30 mg/kg/day i.p.) for 4 days. Echocardiographic measurements were performed at the end of the experiment one week after infarction. The number of animals was 15 in SHAM + veh, 4 in AMI + veh and 3 in AMI + 3i-1000 groups. (**E–H**) The echocardiographic parameters and the mRNA levels in the left ventricular tissue of rats that underwent acute myocardial infarction (AMI) or sham-operation (SHAM) and were treated either with vehicle or compound 3i-1000 (30 mg/kg/day i.p.) for one week. Echocardiographic measurements were performed at the end of the experiment at one week. The number of the animals was 6 in SHAM + veh, 7 in AMI + veh and 8 in AMI + 3i-1000 groups. mRNA levels were measured by RT-PCR and normalised to housekeeping gene 18 S quantified from the same samples. The results are averages ± SEM. *p < 0.05, **p < 0.01, ***p < 0.001 (one-way ANOVA followed by a least significant difference post hoc test).

Acute myocardial infarction (AMI) was induced by ligation of left anterior descending (LAD) coronary artery both in mice and rats. The animals underwent either AMI or SHAM operation and were treated with vehicle (DMSO) or 3i-1000 (30 mg/kg/day i.p.). The mice were treated with 3i-1000 for four days after AMI and followed up to one week. Echocardiographic evaluation showed significant improvement in left ventricular (LV) ejection fraction (EF, Fig. 4A) and fractional shortening (FS, Fig. 4B) with 3i-1000. In addition, a significant attenuation of myocardial structural changes (LV dilatation, wall hypertrophy) was observed, as reflected by the decrease in LV diameters, LV volumes and LV inner diameters in 3i-1000 treated animals (Supplementary Table S1). LV mass and heart weight to body weight ratio were also lower in 3i-1000 treated mice (Supplementary Tables S1 and S2). Furthermore, myocardial infarction associated up-regulation of ANP and BNP gene expression^22^ was significantly decreased with 3i-1000 (Fig. 4C and D), and there was a trend (*p* = 0.052) for the scar size to decrease (Supplementary Table S2). Interestingly the treatment with 3i-1000 had no influence on GATA4 or NKX2-5 gene expression in the left ventricles (Supplementary Table S2). Treatment with 3i-1000 had no significant effect of diastolic function (E’/A’ ratio and LV isovolumic relaxation time, IVRT) (Supplementary Table S1).

In rats, the experimental design was otherwise similar to mice except that the rats were treated with 3i-1000 (30 mg/kg/day i.p.) for one week after AMI. As shown in Fig. 4E and F, ligation of LAD alone caused a modest decrease in left ventricular EF and FS. There was a tendency for the improved cardiac function (Fig. 4E and F) and attenuation of ANP (Fig. 4G) and BNP (Fig. 4H) gene expressions with treatment of 3i-1000. However, these changes were not statistically significant. There was a non-significant improvement in LV dilation (diastolic LV diameter, *p* = 0.081) and LV volume (*p* = 0.084) and significant decrease in diastolic LV inner diameter (*p* = 0.05) in response to administration of 3i-1000 (Supplementary Table S3). In addition, a significant increase of c-kit positive cells in 3i-1000 treated AMI rats compared to vehicle treated animals was noted (Supplementary Table S4).

### Improvement of cardiac function in an experimental model of pressure overload by 3i-1000

**Figure 5:**
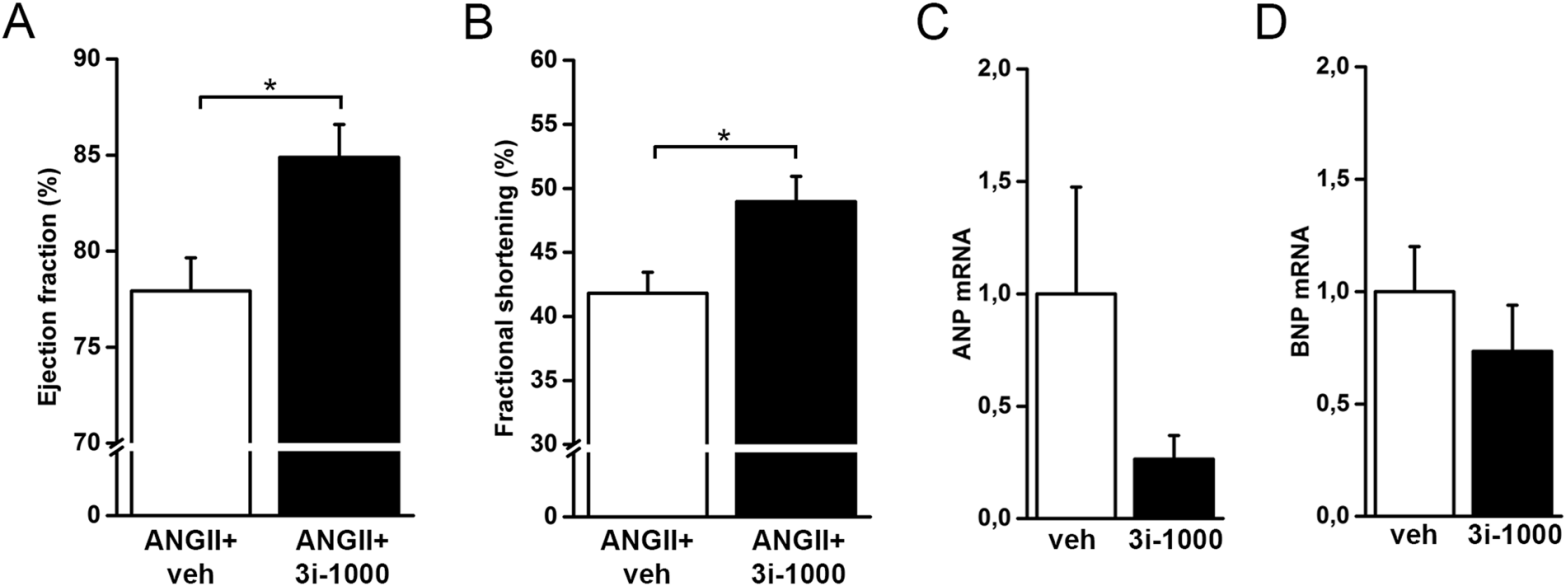
(**A–D**) The echocardiographic parameters and mRNA levels in the left ventricular tissue of rats that were treated with angiotensin II (ANGII, 33.3 µg/kg/h s.c.) and vehicle or ANGII and the compound 3i-1000 (30 mg/kg/day i.p) for two weeks. Echocardiographic measurements were performed at 2 weeks. The number of animals in both groups was 6. mRNA levels were measured by RT-PCR and normalised to housekeeping gene 18 S quantified from the same samples. The results are averages ± SEM. *p < 0.05 (independent samples Student’s *t*-test).

We next evaluated the cardiac effects of 3i-1000 in an experimental model of angiotensin II -mediated hypertension. Angiotensin II infusion in rats increases blood pressure and induces hypertrophic changes in the heart, such as increased ANP expression^23,24^. Angiotensin II was administered via subcutaneously implanted osmotic minipumps (33.3 µg/kg/h) for two weeks, as described previously^24,25^. Interestingly, administration of 3i-1000 at the dose of 30 mg/kg/day i.p. for two weeks significantly increased left ventricular EF (Fig. 5A) and FS (Fig. 5B). There was also a trend for 3i-1000 to decrease left ventricular ANP and BNP expression (Fig. 5C,D). Furthermore, decreases in E’/A’ ratio (*p* = 0.030) and left ventricle diameter (diastolic *p* = 0.032 and systolic *p* = 0.016) and increases in intra ventricular septum (IVS diastole, *p* = 0.044) and heart rate (*p* = 0.001) was noted (Supplementary Table S5), while LV mass did not change significantly (Supplementary Tables S5 and S6).

### Protective effect of 3i-1000 in experimental model of myocardial ischemia reperfusion injury

**Figure 6:**
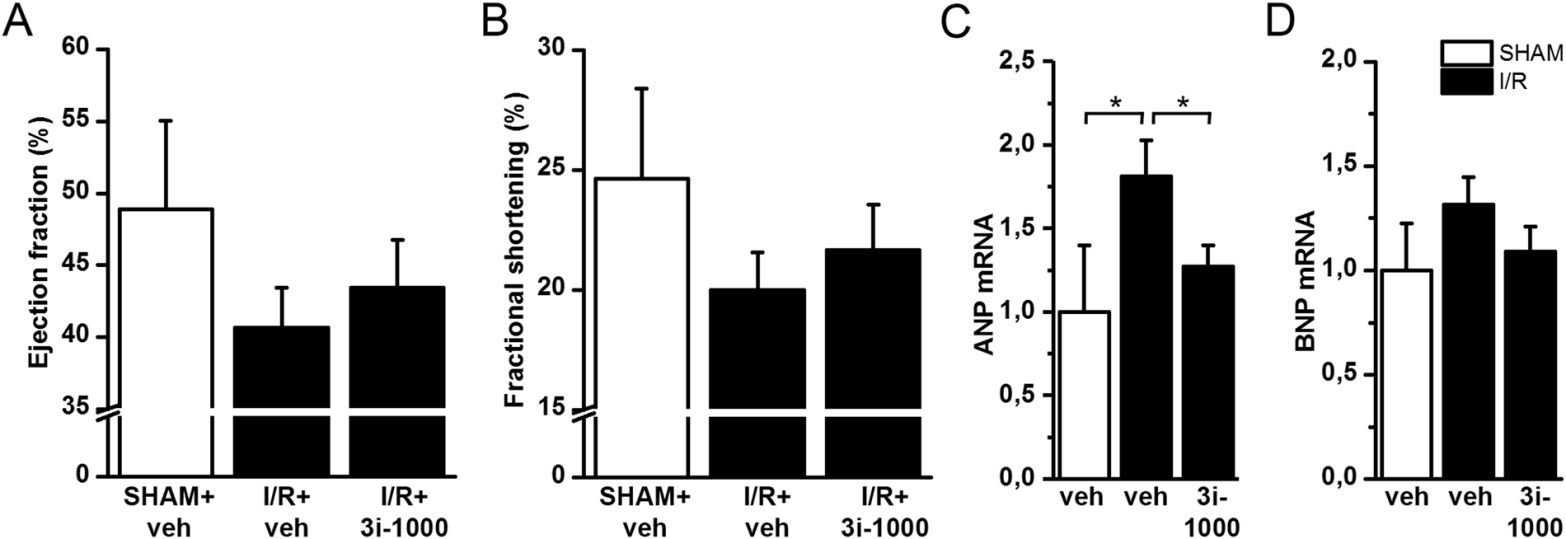
(**A,B**) The left ventricular ejection fraction and fractional shortening analysed by echocardiography in rats treated either with vehicle or compound 3i-1000 (30 mg/kg/day i.p.) for one week before ischemia-reperfusion (I/R) or sham-operation (SHAM). Following 30 min of ischemia, the slipknot was released and the myocardium was re-perfused for 24 h. Echocardiographic measurements were performed at 24 h. The number of the animals was 4 in SHAM+veh, 15 in I/R+veh and 15 in I/R+3i-1000 groups. (**C,D**) The ANP and BNP mRNA levels in the left ventricular tissue. Number of animals was 4 in SHAM+veh, 13 in I/R+veh and 15 in I/R+3i-1000 groups. mRNA levels were measured by RT-PCR and normalised to housekeeping gene 18 S quantified from the same samples. The results are averages ± SEM. *p < 0.05 (one-way ANOVA followed by a least significant difference post hoc test).

To study potential prophylactic cardioprotective effect of 3i-1000, the compound was administered at the dose of 30 mg/kg/day ip. for one week before performing ischemia-reperfusion (I/R) operation in mice. In these experiments, LAD was ligated for 30 min and then re-perfused for 24 h. While 3i-1000 had no significant effect on cardiac function (Fig. 6A and B; Supplementary Table S7), 3i-1000 pre-treatment significantly decreased ANP gene expression (Fig. 6C) induced by I/R injury. A similar, yet non-significant, decrease was observed in BNP expression (Fig. 6D). No changes were observed in GATA4, NKX2-5 or collagen type I alpha 1 chain (COL1A1) gene expression or apoptosis in 3i-1000 pre-treated animals (Supplementary Table S8)

### The effect of intramyocardial delivery of 3i-1000 loaded microparticles in myocardial infarction

**Figure 7:**
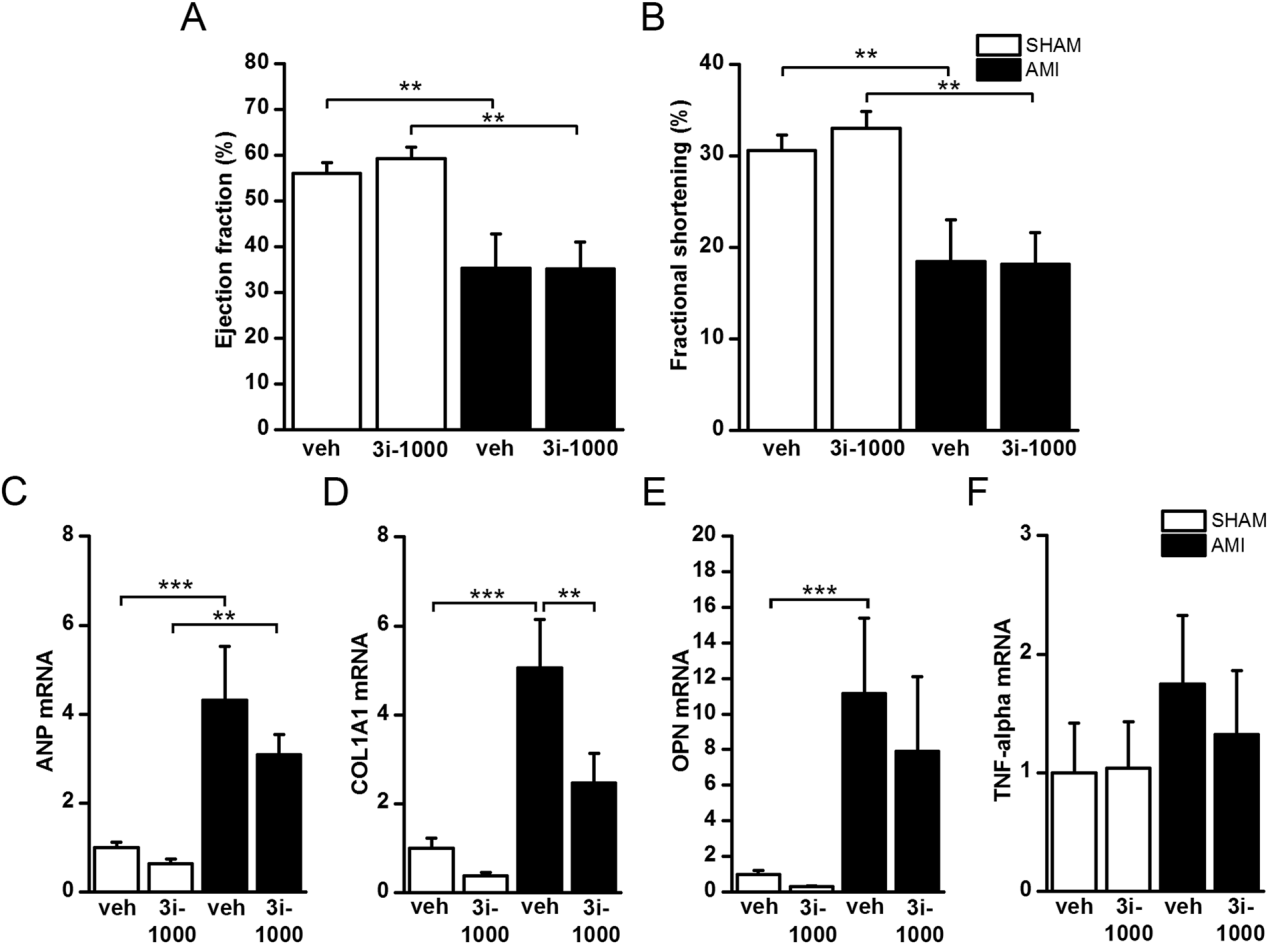
(**A,B**) The echocardiographic parameters and **(C–F)** mRNA levels in the left ventricular tissue of rats that underwent acute myocardial infarction (AMI) or sham-operation (SHAM) and were treated either with vehicle or compound 3i-1000 loaded TOPSi 7 µm particles. Echocardiographic measurements were performed at one week. The number of animals was 7 in SHAM + veh, 7 in SHAM + 3i-1000, 6 in AMI + veh and 7 in AMI + 3i-1000 groups. mRNA levels were measured by RT-PCR and normalised to housekeeping gene 18 S quantified from the same samples. The results are averages ± SEM. *p < 0.05, **p < 0.01, ***p < 0.001 (one-way ANOVA followed by a least significant difference post hoc test).

Since the compound had a short half-life and there was only a tendency for the improved cardiac function with 3i-1000 treatment in rats after AMI, we investigated the local effects of the compound on the left ventricular myocardium using biodegradable and biocompatible porous silicon (PSi) micro- and nanoparticles. We have reported previously that thermally oxidized porous silicon microparticles (TOPSi) can be safely injected intramyocardially in rats after myocardial infarction^26^. The 3i-1000 loaded TOPSi microparticles were injected locally into the left ventricular wall at the same time with LAD ligation in rats. The loading degree of the 3i-1000 TOPSi 7 µM particles was 3.5 ± 1.3%. While cardiac function (Fig. 7A and B; Supplementary Table S9) remained unchanged with intramyocardial delivery of 3i-1000 loaded microparticles at one week, 3i-1000 loaded microparticles significantly decreased COL1A1 gene expression (COL1A1) (Fig. 7D). There was also a tendency for ANP, tumour necrosis factor-alpha (TNF-α) and osteopontin (OPN, also known as SPP1) mRNA levels (Fig. 7C,E and F; Supplementary Table S10) to decrease with intramyocardial delivery of 3i-1000 loaded microparticles.

### Nanoparticles loaded with 3i-1000 modulate left ventricular gene expression

**Figure 8:**
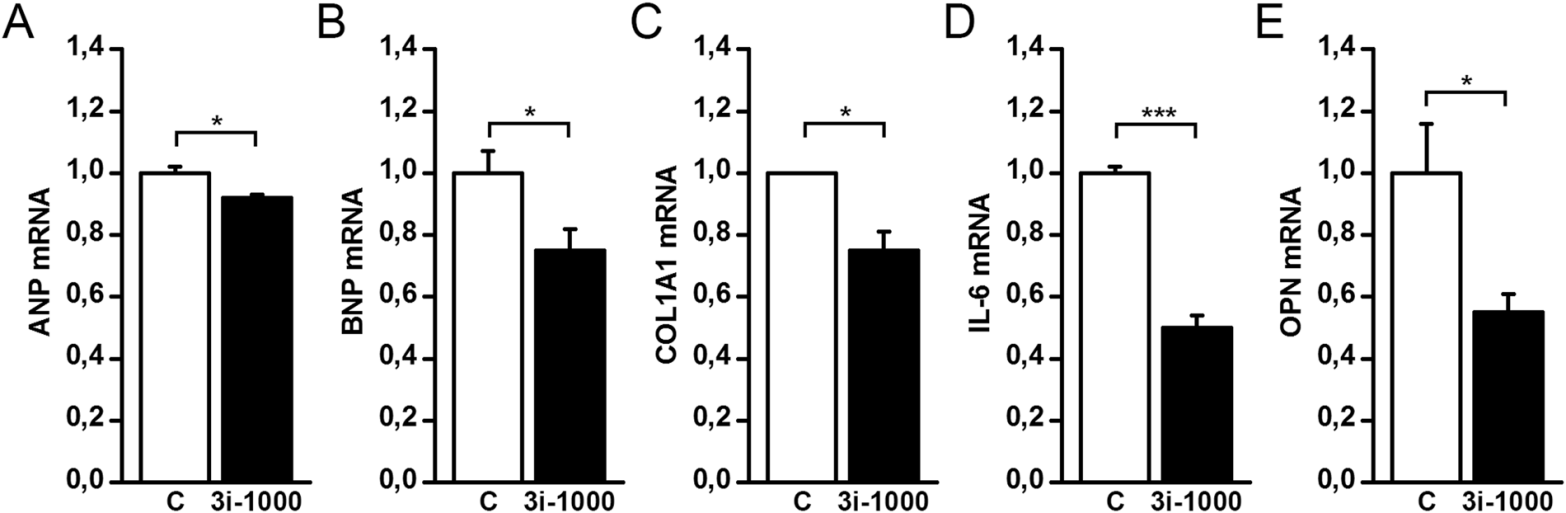
(**A–E**) The mRNA levels in the left ventricular endocardial layer of rats in response to isoprenaline-induced myocardial ischemia after treatment with 3i-1000 loaded nanoparticles. Isoprenaline was injected s.c. 24 h before i.v. administration of the control particles (C) or 3i-1000 loaded particles. The samples were collected 4 h after particle injections. The number of animals was 4–5 in both groups. mRNA levels were measured by RT-PCR and normalised to housekeeping gene 18 S quantified from the same samples. The results are averages ± SEM. *p < 0.05, ***p < 0.001 (independent samples Student’s *t*-test).

Finally, we used circulating biodegradable porous silicon (PSi) nanoparticles functionalised with ANP and loaded with 3i-1000 (labelled C1 in Ferreira *et al*.^27^) to study the effect of the compound in isoprenaline-induced (ISO) myocardial ischemic injury model in rats. We have recently reported that the PSi nanoparticles functionalised with ANP (Un-P-D-ANP nanoparticles) accumulate in the ischemic rat heart, particularly into the endocardial layer of the left ventricle^27^. Isoprenaline, a beta-receptor adrenergic agonist, induces profound myocardial ischemic changes and tachycardia when administered into rats^28^. After single isoprenaline dose of 5 mg/kg s.c., the LV function is impaired at day 1, improving by day 3 after injection^28^. Thus, we injected rats with the isoprenaline dose of 5 mg/kg s.c. and then 24 h later, Un-P-D-ANP nanoparticles loaded with 3i-1000 were injected intravenously (i.v.). As shown in Fig. 8, Un-P-D-ANP nanocarriers loaded with 3i-1000 inhibited significantly induced ANP, BNP, COL1A1, interleukin-6 (IL-6) and OPN gene expressions in the endocardial layer of the ischemic rat heart at 4 h after i.v. injection.

## Discussion

Myocardial infarction and hypertension are leading causes of heart failure and death worldwide^1^. Preservation of contractile function and protection against adverse changes in ventricular architecture (myocardial remodelling) are key factors to limiting progression of this condition to heart failure^2,3^. Consequently, new therapeutic targets are urgently required to achieve this aim. Transcription factors are fundamental regulators of gene expression, and thus have emerged as promising targets for therapeutic manipulation of many diseases^4^. Previously, we have found evidence for functional role of GATA4–NKX2-5 interaction in mediating stretch-response in cardiomyocytes by using rat BNP promoter together with site-specific mutations of GATA and NKX2-5 binding elements^19^. Very recently, we have reported the identification, chemical and biological characterization of small molecules that either inhibit or enhance the GATA4–NKX2-5 transcriptional synergy^20^. Here we show that the most potent inhibitor (3i-1000) of GATA4–NKX2-5 interaction had beneficial effects on cardiac function and gene expression in several experimental models of myocardial ischemia and pressure overload, implicating that modulators of protein–protein interactions of key transcription factors may present a new class of pharmaceuticals for myocardial remodelling and repair.

A core set of evolutionally conserved transcription factors (GATA4, HAND2, MEF2C, NKX2-5 and Tbx5) controls cardiogenesis and heart development^29^. Of these TFs, GATA4 is also a critical regulator of key biological processes in myocardial remodelling and repair^6,7,12,14^. For example, by using adenoviral antisense strategy we observed that decreased GATA4 protein levels inhibit stretch-activated hypertrophy *in vitro*^30^. Conversely, peri-infarct intramyocardial delivery of adenoviral vector expressing GATA4 prevented the adverse post-infarction remodelling through enhanced angiogenesis, decreased apoptosis, and increased stem cell recruitment^11^. On the other hand, the expression of NKX2-5, a co-factor of GATA4 and essential for cardiac development^31^, is up-regulated by hypertrophic stimulus resulting in increased expression of its target genes^4^. Overexpression of NKX2-5 in cultured cardiomyocytes prevents H_2_O_2_- and doxorubicin-induced apoptosis^32,33^, suggesting cardioprotective role also for NKX2-5. Furthermore, the doxorubicin-induced myocardial damage and apoptosis have been shown to be more severe in transgenic mice overexpressing dominant-negative NKX2-5 than in transgenic mice overexpressing wild-type NKX2-5^32^. Finally, cardiac malformations that associate with mutations of GATA4 or NKX2-5 genes may be in part due to disruption of their combinatorial interactions^34^. Based on the crucial role of GATA4 and NKX2-5 in myocardial remodelling and repair processes, we therefore designed drug discovery and development platform and identified compounds possessing either agonistic or antagonistic effect on synergy arising from the protein-protein interactions of GATA4 and NKX2-5^20^.

It is well established that GATA4 and NKX2-5 are key synergistic regulators of ANP gene expression^15^. We have also reported that both GATA4 and NKX2-5 sites are needed for mechanical stretch-activated BNP gene activation^19^. In the present study, we observed that the small molecule inhibiting GATA4–NKX2-5 transcriptional synergy significantly reduced the activation of ANP and BNP gene expression in response to mechanical stretch at micromolar concentrations. The compound also inhibited ANP and BNP gene expression induced by hypertrophic agonist PE. Previously, we have reported that 3i-1000 significantly inhibited the increase in the area of the cardiomyocytes in response to stretching and the endothelin-1 (ET-1) induced hypertrophic response in neonatal rat cardiomyocytes^20^. Moreover, 3i-1000 inhibited GATA4 induced activation of BNP promoter containing the GATA-sites^20^. Taken together, these results indicate that the small molecule 3i-1000 can modulate the hypertrophic response in myocytes *in vitro*, which agrees with the inhibition of the GATA4−NKX2-5 interaction in luciferase reporter gene assay. Of note, another compound (3i-0777), which enhanced GATA4–NKX2-5 transcriptional synergy in luciferase reporter assay^20^, also activated mechanical stretch-induced natriuretic peptide gene expression.

In addition to protein–protein interactions, the transcriptional activity of GATA4 is regulated through post-translational modifications, which affect its DNA binding activity, transactivation and/or localization within cardiac myocytes^5^. The transcriptional activity of GATA4 has been shown to increase in luciferase reporter assays by phosphorylation^35^, acetylation^36^ or sumoylation^37^. The GATA4 phosphorylation of Ser-105 has been shown to be necessary for stress-induced cardiac hypertrophy *in vivo*^38^, and a number of hypertrophic stimuli, such as ET-1, PE and ISO increase GATA4-Ser105 phosphorylation *in vitro*^5^. Our present results show that 3i-1000 significantly decreased PE-induced GATA4 Ser-105 phosphorylation in cardiomyocytes. This finding is consistent with the effect of 3i-1000 on inhibition hypertrophic process activated by mechanical stretch and hypertrophic agonists. Protein kinases shown to catalyse Ser-105-specific phosphorylation of GATA4 in cardiomyocytes are extracellular signal-regulated kinase (ERK) and p38 mitogen-activated protein kinase (p38 MAPK), two members of the MAPK family^5^. In kinase assay, 3i-1000 exhibited no direct activity on these protein kinases involved in the regulation of GATA4 phosphorylation^20^.

Remarkably, our *in vivo* experiments showed that the small molecule inhibitor of GATA4–NKX2-5 interaction has beneficial effects on cardiac function and gene expression in several experimental models of myocardial ischemia and pressure overload. Echocardiographic evaluation showed significant improvement in left ventricular ejection fraction and fractional shortening, and a significant attenuation of myocardial structural changes in 3i-1000 treated mice after myocardial infarction. The compound also improved cardiac function in an experimental model of angiotensin II -mediated hypertension in rats. Moreover, myocardial infarction associated up-regulation of natriuretic peptide gene expression and myocardial ischemia reperfusion injury induced increase in ANP gene expression were significantly decreased by 3i-1000 in mice. Since there was only a tendency for the improved cardiac function and attenuation of ANP and BNP gene expressions with 3i-1000 treatments in rats after AMI, likely due to its rapid metabolism, we investigated the effects of the compound on left ventricular myocardium using biodegradable and biocompatible porous silicon (PSi) micro- and nanoparticles as tools for local administration. When 3i-1000 loaded microparticles were injected intramyocardially in rats, a significant decrease in collagen 1A1 gene expression was noted. Furthermore, circulating PSi nanoparticles functionalised with ANP and loaded with 3i-1000 inhibited significantly ANP, BNP, collagen 1A1, interleukin-6 and osteopontin gene expressions in the endocardial layer of the ischemic rat heart. Previously, we have reported that nanocarriers loaded with 3i-1000 and injected i.v. into rats with isoprenaline induced cardiac injury modulated hypertrophic signalling in the endocardial layer of the rat heart^27^. Overall, all these proof-of-principle experiments indicate significant potential for 3i-1000 to promote myocardial repair after myocardial infarction and other cardiac injuries, and thus, to treat or prevent the development of heart failure. However, further experiment in large animal models, optimization of the properties due to short half-life and rapid metabolisms, as well as a more detailed assessment of the molecular mechanisms of action of 3i-1000 are needed.

To our best of knowledge, this is the first report of a small molecule targeting TF interactions showing beneficial effects on myocardial remodelling in *in vivo* experiments. There are a small number of studies where small molecules have been used to improve cardiac function *in vivo*, yet they require also either cell transplantation or gene delivery. Sadek *et al*.^39^ has identified a cardiogenic small molecule Shz that increases NKX2-5 expression and induces stem cells differentiation into more cardiomyocyte-like cells *in vitro*. The pre-treatment of human mobilised peripheral blood mononuclear cells (M-PBMCs) with Shz prior the injection into the cryo-injured rat heart improved the left ventricular function. It has also been shown that VUT-MK142, a modified cardiogenol C, induces ANP and NKX2-5 expression and promotes cardiovascular progenitor cells differentiation into beating cardiomyocytes *in vitro*^40^. Moreover, intramyocardial injections of GATA4, MEF2C and TBX5 (GMT)-encoding retrovirus and 2 weeks i.p. injections of SB431542 (transforming growth factor-β inhibitor) and XAV939 (glycogen synthase kinase 3β inhibitor) after MI induced improvement of ejection fraction and reduced the scar size by remuscularisation^41^. Previously GMT alone were reported to convert neonatal fibroblasts to cardiomyocyte-like cells *in vitro*^42^. Moreover, forced expression of these three TFs alone (and by adding fourth, HAND2) in mice reprogrammed non-cardiomyocytes into functional cardiac-like myocytes and reduced adverse myocardial remodelling following myocardial infarction^43,44^. Finally, Russel *et al*.^45^ have identified Isx1 as a small molecule capable of enhancing NKX2-5 expression *in vitro*. When this compound was injected for 1 week i.p. in an experimental AMI model in mice, it improved cardiac function at day 7 without reduction in scar size. However, further studies revealed that Isx1 rather acts an activator of G protein-coupled receptor than through transcription factors^46^.

In summary, inventions for the new therapeutics to prevent adverse cardiac remodelling and to promote reverse remodelling are urgently needed^47^. Many current treatments target the neurohumoral activation or reduce the workload of the heart^47,48^. Novel recent strategy is to replace the lost or non-functional cardiac myocytes with stem cells, to induce proliferation of the existing myocytes with cardiogenic factors or miRNAs or induce the cardiac stem cells or fibroblasts to differentiate into cardiac myocytes^49–52^. Our approach here has been to target the key regulators of the hypertrophic responses, i.e., transcription factors and especially GATA4, a critical regulator of key biological processes in myocardial remodelling and repair^6,7,12,14^. In the present study, we investigated in detail the cardiac actions of a small molecule that targets GATA4–NKX2-5 interaction *in vitro*, and report that 3i-1000 can have anti-hypertrophic effects *in vitro* and cardioprotective effects *in vivo*, implicating that modulators of protein–protein interactions of key TFs may present a novel pharmaceuticals for cardiac remodelling and repair.

## Methods

### Plasmids

Three high affinity binding sites for NKX2-5 containing luciferase vector p3xHA and mouse NKX2-5 expressing vector pMT2-NKX2-5 have been described previously^21^. The plasmid expressing the mouse GATA4 (pMT2-GATA4) and the empty pMT2 plasmid were kind gifts from D.B. Wilson (Department of Pediatrics, St. Louis Children’s Hospital, USA)^53^.

### COS-1 cell culture

COS-1 cells were cultured in Dulbecco’s modified Eagle’s medium (DMEM) supplemented with 10% foetal bovine serum (FBS), 100 U/ml of penicillin, and 100 µg/ml of streptomycin. The COS-1 cells were kind gift from Dr. Jukka Hakkola (University of Oulu, Finland)^54^. All cell cultures were maintained at 37 °C in humidified atmosphere with 5% CO_2_.

### Luciferase reporter assay

GATA4–NKX2-5 transcriptional synergy reporter assay was performed as described previously^20^. Briefly, COS-1 cells were cultured on 48 or 96-well plates, 35 000 cells per well or 14 000 cells per well, respectively. Cells were co-transfected with 250 ng or 100 ng of p3xHA luciferase vector and 62.5 ng or 25 ng of both expression plasmids pMT2-GATA and pMT2-NKX2-5 or empty pMT2 per well at a 3:1 Fugene 6 Transfection Reagent (Promega):DNA ratio. Six hours later transfection reagent was removed and 3i-1000 was added into cells in DMEM containing 0.1% DMSO (Sigma). New media with the compound was changed to the cells, except on 96-well plate format, at 12 h from transfections. The cells were exposed to 3i-1000 for 24 h and then the cells were lysed with Passive Lysis Buffer (E194A, Promega). The samples were processed with Luciferase Assay System (E1500, Promega) and measured with a luminometer (Luminoskan RS, Labsystems or Victor2, Perkin Elmer). The compound was tested in three or eight technical replicates in two independent experiments.

### Induction of hypertrophic response by mechanical stretch and phenylephrine in neonatal rat cardiomyocytes

Primary cultures of neonatal rat ventricular myocytes were prepared from 2- to 4-day-old Sprague-Dawley rats, as described previously^20,27,55^. For stretch experiments, primary cardiomyocytes were cultured on collagen I-coated elastomere plates with flexible bottom (Bioflex, Flexcell) in density of 0.15 million cells per cm^2^. Stretch was introduced to attached cardiomyocytes by a computer-controlled vacuum suction with Flexercell Strain Unit FX-3000 (Flexcell), as described previously^30^. Frequency of cyclic stretch was 0.5 Hz with pulsation of 10–25% elongation of cells for 24 h. Small molecules 3i-1000 and 3i-0777 in supplemented CSFM media containing 0.1% DMSO were added into the cells 1 h before starting the mechanical stretching. For the control samples, the cells were plated and cultured similarly on collagen I flexible bottom culture plates without stretching.

For phenylephrine experiments, primary cardiomyocytes were cultured on normal cell well plates (BD Falcon) at the density of 0.13–0.17 million cells per cm^2^. On the third day on culture, the cells were first exposed to 3i-1000 in supplemented CSFM media containing 0.1% DMSO and then 1 h later, 100 μM phenylephrine (Sigma) was added into the media for 24 h.

### Isolation and analysis of RNA

Total RNA from primary cardiomyocytes was isolated with TRIzol reagent (Invitrogen), following the manufacturer’s protocol by using the Phase Lock Gel system (Eppendorf AG). In the *in vivo* experiments, the apex of the LV was immersed in liquid nitrogen and stored at −70 °C for further analysis. The LV tissue was grinded to powder in liquid nitrogen and total RNA was isolated by the guanidine thiocyanate–CsCl method^56^. For real-time polymerase chain reaction (RT-PCR) analyses, cDNA was synthesised from total RNA with a First-Strand cDNA Synthesis Kit (GE Healthcare Life Sciences), following the manufacturer’s protocol. RNA was analysed by RT-PCR on an ABI 7300 sequence detection system (Applied Biosystems) using TaqMan chemistry. The sequences of the forward and reverse primers and fluorogenic probes for RNA detection are shown in Supplementary Table S12. The results were quantified using ΔΔC_T_ method and normalised to housekeeping gene 18 S quantified from the same samples.

The total RNA of the isoprenaline-induced myocardial ischemia experiments was isolated from the endocardial layer of LV as described earlier^27^. The reverse transcriptase reactions and real-time PCR analysis was performed using Fluidigm TaqMan 48.48 array by the Biomedicum Functional Genomics Unit (FuGU), Finland. Briefly, the quantity and quality of the RNA was analysed and 50 ng of total RNA was used for production of pre-amplified cDNA according to Real-Time PCR Analysis User Guide (PN 68000088 K1, Fluidigm). The TaqMan qPCR analysis on the 48.48 array was performed according to the Gene Expression protocol with the 48.48 IFC Using Fast TaqMan Assays (Biomark HD Only) (PN 100-2637 E1, Fluidigm). The qPCR conditions were 95 °C for 60 sec, 40 repeats of 95 °C for 5 sec and 60 °C for 20 sec. The following predesigned TaqMan probes from Thermo Fisher Scientific were used in the assay: 18 s (4352930E), BNP (Rn00580641_m1), ANP (Rn00664637_g1), TGFB1 (Rn00572010_m1), COL1A1 (Rn01463848_m1), IL-6 (Rn01410330_m1), TNF-alpha (Rn01525859_g1), OPN (Rn00681031_m1), NKX2-5 (Mm01309813_s1) and GATA4 (Rn01530459_m1). From each animal three to four technical replicates were analysed and average of these replicates, as well as quantified 18 S measured from the same samples, were used to calculate ΔΔC_T_. Grubbs’ test was used to detect the outliers.

### Protein extraction

The nuclear and cytosolic proteins were extracted from cultured cardiomyocytes with modified method of Schreiber *et al*.^57^. The cells were washed and scraped with phosphate-buffered saline (PBS). After centrifugation the cell pellets were re-suspended in low salt buffer consisting of 10 mM HEPES (4-(2-hydroxyethyl)-1-piperazineethanesulfonic acid), 10 mM KCl, 0.1 mM EDTA and 0.1 mM EGTA supplemented with protease (P8340, Sigma) and phosphatase (P0044, Sigma) inhibitor cocktails (1:100 volume). The suspensions were then allowed to swell on ice. Next, membrane proteins were solubilised and isolated by adding 10% Igepal CA-630 detergent and vortexing vigorously followed by centrifugation. The supernatants were collected as the cytosolic fragments. The pellets were re-suspended in high salt buffer containing 20 mM HEPES, 0.4 M NaCl, 1 mM EDTA and 1 mM EGTA, with supplements similar to those previously and then rocked for 15 min. The supernatant was collected by centrifugation as the nuclear fragment. The entire procedure was carried out at + 4 °C. Protein concentrations were determined with the Bio-Rad Protein Assay.

### Western blotting

Protein samples (20–40 g) were loaded on SDS-PAGE and transferred to nitrocellulose filters. After blocking the nonspecific background in 5% non-fat milk, nitrocellulose membranes were incubated at +4 °C overnight with anti-GATA4 (sc-1237) or anti-Lamin B (sc-6216) (Santa Cruz Biotechnology). To detect phosphorylated GATA4 the membrane was blocked in 5% non-fat milk overnight + 4 °C and incubated with anti-phospho-GATA4 (Ser105) (44-948, Invitrogen) for 2 h at room temperature. After washing, the filters were incubated for 1 hour with an HRP-conjugated anti-rabbit, anti-mouse or anti-goat secondary antibody. The protein amounts were detected by enhanced chemiluminescence with ECL Plus reagents (RPN2132, Amersham Biosciences) followed by digitalization of chemiluminescence with Luminescent Imager Analyzer LAS-3000 (Fujifilm) and analyzing with Quantity One software (Bio-Rad Laboratories). For infrared detection, the secondary fluorescent antibodies were Alexa Fluor 680 from Invitrogen (Life Technologies Ltd, Paisley, UK) and IRDye 800 from Rockland Immunochemicals (Gilbertsville, PA, USA). Antibody binding was detected by the Odyssey Infrared Imaging System (LI-COR, Lincoln, NE). For a second immunoblotting, the membrane was stripped for 30 min at + 60 °C in stripping buffer (62.5 mM Tris pH 6.8, 2% SDS and 100 mM β -mercaptoethanol).

### Microparticle fabrication, drug loading and characterization

Thermally oxidized porous silicon microparticles (TOPSi) were prepared by electrochemical anodization, as previously described^58^. Briefly, free-standing multilayer PSi films were electrochemically anodized from monocrystalline *p* + Si < 100 > wafers with a solution of 1:1 (v/v) hydrofluoric acid (38%)‒ethanol (EtOH), using a constant etching current. The porous films were then lifted from the Si substrates by an abrupt increase of the etching current. The stabilisation was done by thermally oxidizing the multilayer films in ambient air for 2 h at 300 °C, followed by dry milling, sieving and centrifugation. The physicochemical characterization of the vehicle-loaded particles, including average pore diameter (12.7 ± 0.1 nm), specific surface area (193 ± 2 m^2^/g), total pore volume (0.61 ± 0.01 cm^3^/g), average size distribution and chemical surface characterization, was determined as described previously^26^.

The drug loading was performed with immersing the TOPSi microparticles (10 mg) in a solution of 3i-1000 (10–20 mg/ml in EtOH) during 90 min under stirring. The particles were centrifuged (16100 *g*, 4 min) and dried at 60 °C for 1 h, yielding 3i-1000-TOPSi microparticles. The 3i-1000-TOPSi (1 mg) microparticles were added to 2 ml of EtOH and allowed to release the 3i-1000 content for 45 min. The supernatants were analysed by high-performance liquid chromatography (HPLC) using an Agilent 1100 series HPLC system (Agilent Technologies), a Gemini−NX 3 µm C18 110 Å reversed phase column (100 × 4.6 mm, Phenomenex, USA) as a stationary phase, and a mix of 0.1% phosphoric acid (PA) (pH 3.0) and MeOH (ratio of 60:40 v/v) as a mobile phase. The flow rate was set at 0.9 ml/min, the injection volume 50 μl, and the wavelength was 280 nm. The loading degree (LD) was calculated based on the equation (1).

1 $${LD}\,( \% )=\frac{{\rm{mass}}\,{\rm{of}}\,{\rm{loaded}}\,{\rm{C}}1\,(\mu {\rm{g}}/{\rm{ml}})\times \mathrm{volume}\,({\rm{ml}})}{\,\mathrm{total}\,{\rm{mass}}\,{\rm{of}}\,{\rm{drug}}-{\rm{loaded}}\,{\rm{particles}}\,(\mu {\rm{g}})\,}\times 100$$

### Nanoparticle fabrication, drug loading and characterization

The ANP functionalized nanoparticles (Un-P-D-ANP), their fabrication, functionalization, characterization and drug loading have been described in detail previously^27,59^. The physicochemical characterization of the functionalized nanoparticles, including average pore diameter (10.4 ± 0.2 nm), specific surface area (281 ± 31 m^2^/g), total pore volume (0.75 ± 0.04 cm^3^/g), as well as their average hydrodynamic diameter (229 ± 1 nm) and polydispersity index (0.15 ± 0.03) were determined as reported previously^27^.

### Myocardial infarction in mice

Myocardial infarction in mice was produced by coronary artery ligation using a technique, which does not require ventilation^60^. Briefly, 8-10 weeks old male C57BL/6 mice underwent either surgical procedure with ligation of left anterior descending coronary artery (LAD) or same operation without ligation of LAD (SHAM operation). The mice were treated preoperatively with carprofen 5.0 mg/kg s.c. All animals were monitored after the surgery and received buprenorphine 0.1 mg/kg within 6 h after surgery. Carprofen and buprenorphine were administered s.c. one day after operation and then in drinking water for 3 days. The 3i-1000 compound was administered twice daily (30 mg/kg/day i.p.), the first dose given was after the operation. The echocardiography was performed at 3 days and 1 week after operation.

### Myocardial infarction in rats

Myocardial infarction in male Sprague-Dawley rats (250-300 g) was produced by ligation of the LAD during medetomidine hydrochloride and ketamine hydrochloride anaesthesia as previously described^26^. Buprenorphine hydrochloride 0.1 mg/kg twice daily and carprofen 5 mg/kg once daily were administered s.c. for three days as a post-operative analgesia. The sham‒operated rats underwent the same surgical procedure without the ligation of LAD. The 3i-1000 compound was administered twice (30 mg/kg/day i.p.), the first dose given after the operation. In microparticle experiments, either control or 3i-1000 loaded TOPSi 7 μm particles were administered by direct injection into the anterior wall of the LV before the ligation of LAD. The TOPSi particle delivery to the sham–operated hearts was performed using the same technique without the ligation of LAD. At one week post-infarction the echocardiography was performed, the rats were decapitated and LV tissues were collected for further analysis.

### Angiotensin II-induced hypertension

Angiotensin II (33.3 μg/kg per hour) was administered via subcutaneously implanted osmotic minipumps (Alzet model 2002, Scanbur) for 2 weeks to male Sprague-Dawley rats (weighing 250–300 g). The minipumps were installed through neck incision under isoflurane anaesthesia. The 3i-1000 compound was administered at the dose of 30 mg/kg/day i.p. for 2 weeks. The echocardiography was performed before decapitation.

### Ischemia reperfusion injury

Surgical procedure was performed as previously described^60^. Briefly, 8-10 weeks old C57BL/6 male mice were anesthetised with 2% isoflurane inhalation. The heart was exposed and exteriorised through a left thoracotomy at the level of the fifth intercostal space. A slipknot was made around the LAD 1–2 mm from its origin with a 6–0 silk suture. Sham-operated animals were subjected to the same surgical procedures except that the suture was passed under the LAD, but was not tied. Following 30 min of ischemia, the slipknot was released and the myocardium was re-perfused for 24 h. A single dose of buprenorphine (0.3 mg/kg s.c.) was administered for pain treatment. The compound 3i-1000 was administered i.p. at a dose of 30 mg/kg/day for 1 week before ischemia/reperfusion operation. At the end of the experiment, animals were sedated with isoflurane and the echocardiography was performed.

### Isoprenaline induced myocardial ischemia

Myocardial ischemia induced with isoprenaline injection as well as the i.v. delivery of PSi nanoparticles has been described in detail previously^27^. Shortly, 8–9 weeks old male Wistar rats were injected 5 mg/kg isoprenaline s.c. After 24 h, PSi nanoparticles were injected i.v. under isoflurane anaesthesia. The animals were euthanased with CO_2_ at 4 h. The right ventricle was removed and the left ventricle was placed between wide end forceps and frozen in liquid nitrogen to straighten the tissue for endocardium to epicardium separation.

### Echocardiography

Transthoracic echocardiography analysis was performed as previously described^61^. The rats were sedated with isoflurane or in terminal anaesthesia with ketamine (50 mg/kg i.p.) and xylazine (10 mg/kg i.p.) and the mice with isoflurane. All the measurements were made from three subsequent cycles and calculated as mean of these three measurements by trained sonographer (Z.Sz.) blinded to the treatments.

### Plasma concentrations and metabolite profile of study compound

The plasma concentrations and metabolite profile of 3i-1000 were evaluated following a single administration of 10 mg/kg of compound i.p. into male Sprague-Dawley rats weighing 250–300 g. The blood samples were collected into lithium heparin Microvette tubes from tail vein at 0.5 h, 2 h and 6 h after the dosing. Tubes were centrifuged 10 min 1300 *g* at +4 °C and the plasma samples were further analysed by Novamass, Finland, by acquiring data with a Waters LCT Premier XE time-of-flight (TOF) mass spectrometer (Waters Corp.).

### Immunohistochemistry

At end of experiments, the animals were euthanized and the left ventricles were dissected, weighed and prepared half horizontally. The base was used for histological staining and fixed in 10% neutral buffered formalin for 1–2 days, embedded in paraffin, cut into 5 µm-sections from the infarction area and mounted on slides. Sections were deparaffinised in xylene and rehydrated in graded ethanol. To detect apoptotic cells *in situ* labelling of the 3′-ends of the DNA fragments generated by apoptosis-associated endonucleases was performed using the ApopTag *in situ* apoptosis detection kit (Chemicon), as previously described^62^. Masson’s trichrome technique was used to define the extent of fibrosis (fibrotic area/total LV area) in the LV. To identify cells undergoing division, immunohistochemical labeling of nuclear Ki-67 was performed with monoclonal mouse anti-rat Ki-67 (clone MIB-5, M7248, Dako Denmark A/S) at 1:25 dilution. Antibody for c-kit (rabbit polyclonal IgG, sc-168, Santa Cruz Biotechnology) at 1:1500 dilution was used to stain stem-cell like cells in the myocardium. The secondary antibodies and visualisation reagents were included to Dako REAL EnVision Detection System (K5007, Dako Denmark A/S). Finally, the samples were counterstained with haematoxylin. Immunohistological analysis was performed by using a light microscope (Nikon Eclipse 50i). Fibrosis was measured by the Nikon NIS-Elements BR 2.30 program from five representative high-power fields from the infarcted myocardium. All measurements were performed blinded by persons, who were not aware of the treatments.

### Statistics

Results are expressed as mean ± standard error of the mean (SEM) or standard deviation (SD). Statistical analyses were performed using SPSS Statistics 21 (IBM). Statistical significance was evaluated by one-way analysis of variance (ANOVA) followed by a least significant difference (LSD) post hoc test for multiple comparisons. To determine the statistical difference between two groups, the independent samples t-test was used. A probability value of *p* < 0.05 was considered statistically significant.

### Ethics

Animal experiments were carried out in accordance with the 3 R principles of the EU directive 2010/63/EU governing the care and use of experimental animals, and following local laws and regulations [Finnish Act on the Protection of Animals Used for Scientific or Educational Purposes (497/2013, Government Decree on the Protection of Animals Used for Scientific or Educational Purposes (564/2013)]. The protocols were approved by the national Animal Experiment Board of Finland (ESAVI-2028-041007-2014).

### Data availability

The datasets generated during the current study are available from the corresponding author on reasonable request.

## Electronic supplementary material

Supplementary Information
