# Supplementary material for: Cardiac Actions of a Small Molecule Inhibitor Targeting GATA4–NKX2-5 Interaction
Source: Sci Rep. 2018 Mar 15;8:4611. doi: 10.1038/s41598-018-22830-8 (PMC5854571; doi:10.1038/s41598-018-22830-8)
Supplement: Supplementary file 1 — Supplementary Information [file 41598_2018_22830_MOESM1_ESM.pdf]

Supplementary data

Cardiac Actions of a Small Molecule Inhibitor Targeting GATA4–NKX2-5 Interaction

Sini M. Kinnunen[1,2,+], Marja Tölli[2,+], Mika Välimäki[1,2], Erhe Gao[3], Zoltan Szabo[2], Jaana Rysä[4], Mónica P.A. Ferreira[5], Pauli Ohukainen[6], Raisa Serpi[2], Alexandra Correia[5], Ermei Mäkilä[7], Jarno Salonen[7], Jouni Hirvonen[5], Hélder A. Santos[5,8], Heikki Ruskoaho[1, 2,*]

[1] *Drug Research Program, Division of Pharmacology and Pharmacotherapy, University of Helsinki, Helsinki, Finland*

[2] *Department of Pharmacology and Toxicology, Institute of Biomedicine, University of Oulu, Oulu, Finland*

[3] *Lewis Katz School of Medicine at Temple University, Philadelphia, Pennsylvania, United States of America*

[4] *School of Pharmacy, Faculty of Health Sciences, University of Eastern Finland, Kuopio, Finland*

[5] *Drug Research Program, Division of Pharmaceutical Chemistry and Technology, Faculty of Pharmacy, University of Helsinki, Helsinki, Finland*

[6] *Computational Medicine, Faculty of Medicine, University of Oulu and Biocenter Oulu, Oulu, Finland*

[7] *Laboratory of Industrial Physics, Department of Physics and Astronomy, University of Turku, Turku, Finland*

[8] *Helsinki Institute of Life Sciences (HiLIFE), University of Helsinki, Helsinki, Finland*

[*]Corresponding author: Heikki Ruskoaho, Division of Pharmacology and Pharmacotherapy, Faculty of Pharmacy, University of Helsinki, P.O.Box 56, FI-00014 Helsinki, FINLAND

email: heikki.ruskoaho@helsinki.fi

[+]authors contributed equally to this work

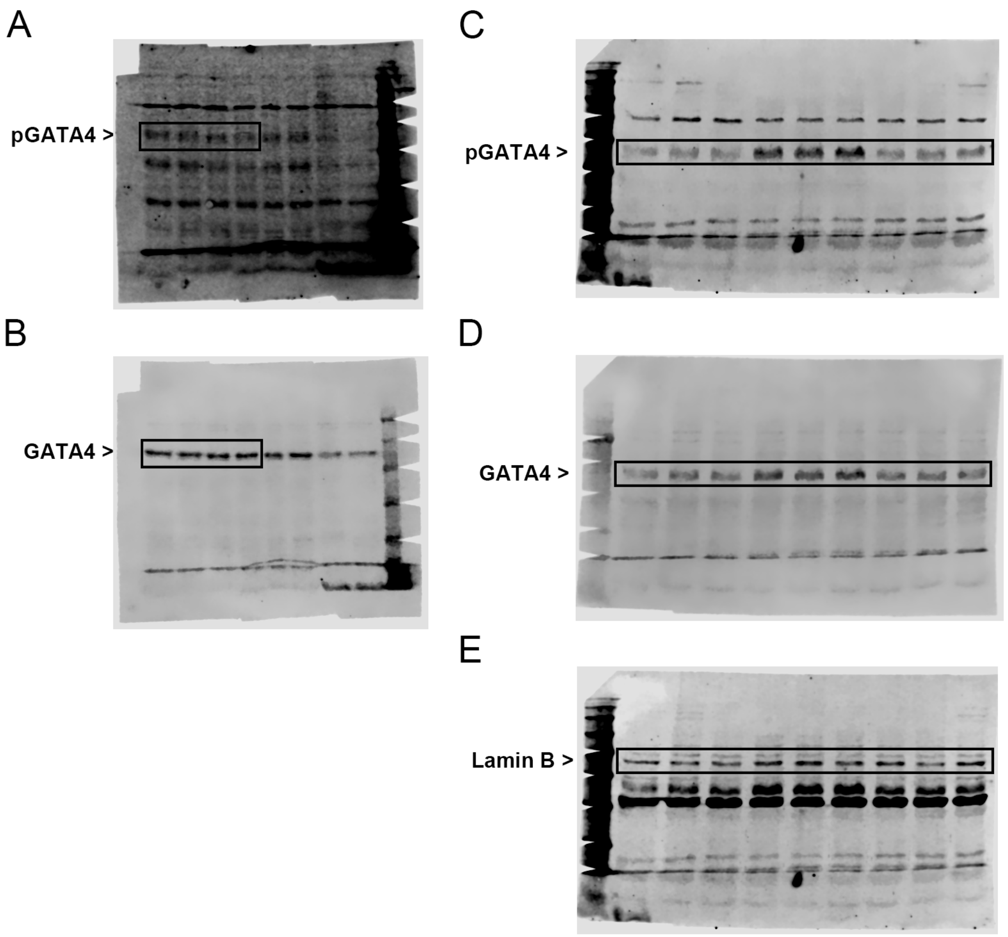

Figure S1. The original western blot images for Figure 3. (**A, B**) The bands shown in Figure 3A and (**C-E**) the bands shown in Figure 3B are circled by a rectangle.

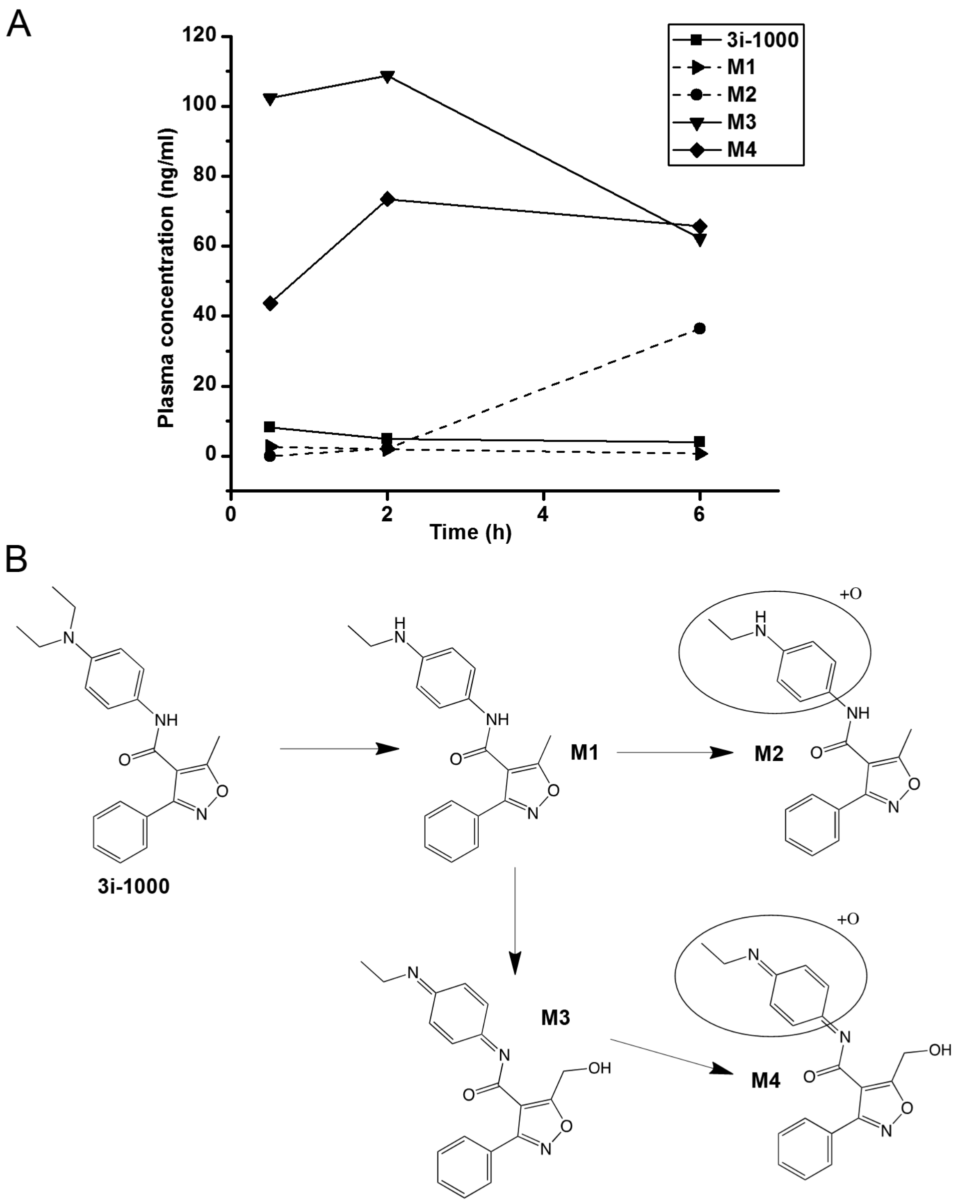

Figure S2. Pharmacokinetics and metabolic profile of 3i-1000. (**A**) Plasma concentrations of compound 3i-1000 and its metabolites M1-M4. A single dose of compound 3i-1000 (10 mg/kg) was administered i.p. in rats and blood samples were collected from tail vain. The plasma samples from three animals were pooled and the compound concentrations were analysed from by LC-TOF mass spectrometer. (**B**) Compound structure of 3i-1000 and the most abundant metabolites.

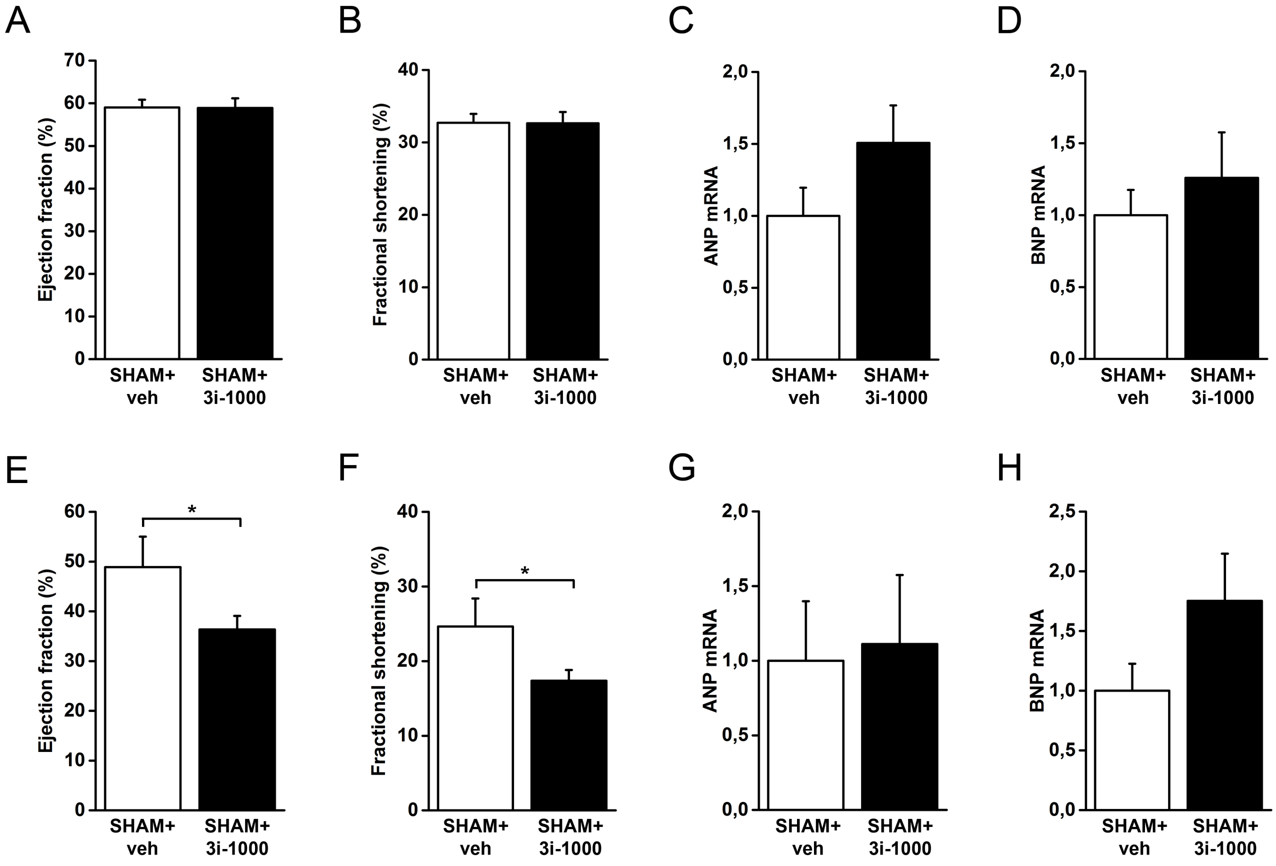

Figure S3. (A-D). The echocardiographic parameters and the mRNA levels in the left ventricular tissue of rats that underwent sham-operation (SHAM) in acute myocardial infarction experiment. Rats were treated either with vehicle (veh, DMSO) or compound 3i-1000 (30 mg/kg/day i.p.) for one week. Echocardiographic measurements were performed at the end of the experiment at one week. The number of the animals was 6 in SHAM+veh and 6 in SHAM+3i-1000. mRNA levels were measured by RT-PCR and normalised to housekeeping gene 18S quantified from the same samples. The results are averages ± SEM. **(E-H).** The echocardiographic parameters and the mRNA levels in the left ventricular tissue of rats that underwent sham-operation in ischemia-reperfusion experiment. Rats where treated either with vehicle or compound 3i-1000 (30 mg/kg/day i.p.) for one week before sham-operation. Sham-operated animals were subjected to the same surgical procedures than animals in the ischemia-reperfusion group except that the suture was passed under the LAD, but was not tied. Following 30 min of non-ischemia, the slipknot was released and the myocardium was still perfused for 24 h. Echocardiographic measurements were performed at 24 h. mRNA levels were measured by RT-PCR and normalised to housekeeping gene 18S quantified from the same samples. The number of the animals was 4 in SHAM+veh and 3 in SHAM+3i-1000 group. The results are averages ± SEM. *$p<0.05$ (one-way ANOVA followed by a least significant difference post hoc test).

Table S1. The echocardiographic parameters of mice that underwent acute myocardial infarction (AMI) or sham-operation (SHAM) and were treated either with vehicle (V) or compound 3i-1000 (30 mg/kg/day i.p.) for 4 days. Echocardiographic measurements were performed at the end of the experiment one week after infarction. The number of animals in each group is shown in brackets. The results are averages ± SEM. Significance (p) was calculated by one-way ANOVA followed by a least significant difference post hoc test. Comparison † SHAM+V vs. AMI+V; ‡ AMI+V vs. AMI+3i-1000. IVRT, isovolumic relaxation time; d, diastolic; s, systolic; BPM, beats per minute; LV, left ventricle; LVID, left ventricular internal diameter; LVPW, left ventricular posterior wall.

	SHAM+V (15)		AMI+V (4)		†	AMI+3i-1000 (3)		‡
	Average	±SEM	Average	±SEM	p	Average	±SEM	
E' (mm/s)	-19,60	1,42	-14,06	2,91	0,081	-23,51	1,42	0,032
A' (mm/s)	-22,51	1,03	-13,29	3,21	0,001	-18,27	1,49	0,151
E'/A'	0,89	0,07	1,21	0,27	0,096	1,30	0,14	0,708
IVRT (ms)	15,64	1,05	21,49	2,43	0,018	15,74	0,74	0,076
Diameter;d (mm)	4,12	0,08	5,29	0,28	0,000	4,53	0,54	0,040
Diameter;s (mm)	3,18	0,09	4,83	0,29	0,000	3,82	0,62	0,019
Ejection Fraction (%)	46,22	2,09	19,19	1,83	0,000	34,62	6,91	0,022
Fractional Shortening (%)	22,99	1,30	8,77	0,85	0,000	16,56	3,47	0,046
Heart Rate (BPM)	447,73	13,01	492,45	14,30	0,095	499,14	11,48	0,848
LV Mass (mg)	107,17	3,88	186,32	18,65	0,000	127,88	18,87	0,003
Volume;d (µl)	75,44	3,28	136,33	16,24	0,000	97,80	27,97	0,042
Volume;s (µl)	41,00	2,57	110,94	15,02	0,000	67,80	26,93	0,015
LVID;d (mm)	4,08	0,08	5,28	0,32	0,000	4,43	0,51	0,030
LVID;s (mm)	3,16	0,10	4,83	0,32	0,000	3,72	0,60	0,014
LVPW;d (mm)	0,74	0,02	0,71	0,09	0,491	0,76	0,07	0,508
LVPW;s (mm)	1,00	0,03	0,84	0,07	0,033	0,91	0,13	0,456

Table S2. The mRNA and histological measurements of mice that underwent acute myocardial infarction (AMI) or sham-operation (SHAM) and were treated either with vehicle (V) or compound 3i-1000 (30 mg/kg/day i.p.) for 4 days. The left ventricular tissues were analysed at the end of the experiment one week after infarction. From SHAM+V group, 8 animals were randomly selected for mRNA measurements and 5 animals for histological analysis. The number of animals in other groups is shown in brackets. The results are averages ± SEM. Significance (p) was calculated by one-way ANOVA followed by a least significant difference post hoc test. Comparison † SHAM+V vs. AMI+V; ‡ AMI+V vs. AMI+3i-1000. ANP, atrial natriuretic peptide; BNP, B-type natriuretic peptide; COL1A1, collagen 1A1; HW/BW, heart weight to body weight.

	SHAM+V		AMI+V (4)		†	AMI+3i-1000 (3)		‡
	Average	±SEM	Average	±SEM	p	Average	±SEM	p
ANP	1,00	0,33	18,27	1,24	0,000	10,92	4,58	0,024
BNP	1,00	0,24	6,43	1,58	0,000	3,16	0,57	0,034
GATA4	1,00	0,12	0,85	0,12	0,453	0,59	0,23	0,307
NKX2-5	1,00	0,11	0,83	0,11	0,393	0,51	0,23	0,191
COL1A1	1,00	0,35	4,58	0,69	0,002	2,43	1,61	0,091
Fibrosis	1,00	0,62	26,61	4,17	0,001	24,70	2,67	0,690
Apoptosis (tunel)	2,44	0,39	3,50	0,85	0,231	4,67	0,57	0,246
Scar	6,61	3,59	37,57	2,41	0,000	26,47	4,56	0,052
Ki67+ cells	0,76	0,25	1,35	0,31	0,330	1,73	0,84	0,571
c-kit+ cells	3,11	1,21	11,11	7,84	0,306	13,20	7,25	0,808
HW/BW	4,95	0,10	7,54	0,46	0,000	5,69	0,38	0,000

Table S3. The echocardiographic parameters of rats that underwent acute myocardial infarction (AMI) or sham-operation (SHAM) and were treated either with vehicle or compound 3i-1000 (30 mg/kg/day i.p.) for one week. Echocardiographic measurements were performed at the end of the experiment at one week. The number of the animals in each group is shown in brackets. The results are averages ± SEM. Significance (p) was calculated by one-way ANOVA followed by a least significant difference post hoc test. Comparison † SHAM+V vs. AMI+V; ‡ AMI+V vs. AMI+3i-1000. IVRT, isovolumic relaxation time; d, diastolic; s, systolic; BPM, beats per minute; LV, left ventricle; IVS, intra ventricular septum; LVID, left ventricular internal diameter; LVPW, left ventricular posterior wall.

	SHAM+V (6)		AMI+V (7)		†	AMI+3i-1000 (8)		‡
	Ave	±SEM	Ave	±SEM	p	Ave	±SEM	p
E' (mm/s)	-40,71	4,31	-38,23	4,70	0,702	-34,02	4,79	0,487
A' (mm/s)	-20,95	1,33	-20,54	2,21	0,940	-21,55	1,67	0,844
E'/A'	1,99	0,28	1,91	0,17	0,797	1,59	0,20	0,304
IVRT (ms)	22,90	0,99	25,66	1,14	0,169	24,26	1,73	0,446
Diameter;d (mm)	8,32	0,17	9,46	0,25	0,009	8,79	0,35	0,081
Diameter;s (mm)	5,61	0,20	7,19	0,42	0,013	6,47	0,52	0,200
Ejection Fraction (%)	59,04	1,80	44,68	6,24	0,042	49,94	5,01	0,405
Fractional Shortening (%)	32,71	1,25	24,02	3,80	0,048	27,09	3,17	0,436
Heart Rate (BPM)	266,27	3,49	282,97	8,06	0,095	267,19	6,45	0,090
LV Mass (mg)	921,41	95,47	1198,41	129,44	0,084	1057,56	86,55	0,334
Volume;d (μl)	377,53	16,49	501,74	28,21	0,008	430,29	37,07	0,084
Volume;s (μl)	155,69	12,33	278,98	36,54	0,013	226,89	39,78	0,235
IVS;d (mm)	1,39	0,16	1,36	0,12	0,914	1,52	1,19	0,487
IVS;s (mm)	2,08	0,21	1,83	0,26	0,472	2,05	0,28	0,506
LVID;d (mm)	8,20	0,17	9,39	0,22	0,006	8,64	0,34	0,050
LVID;s (mm)	5,55	0,18	7,17	0,43	0,010	6,38	0,49	0,152
LVPW;d (mm)	1,51	0,08	1,49	0,10	0,879	1,52	0,12	0,783
LVPW;s (mm)	2,16	0,09	2,10	0,17	0,830	2,09	0,27	0,962

Table S4. The mRNA and histological measurements of rats that underwent acute myocardial infarction (AMI) or sham-operation (SHAM) and were treated either with vehicle or compound 3i-1000 (30 mg/kg/day i.p.) for one week. The left ventricular tissues were analysed at the end of the experiment at one week. The mRNA measurements were performed to all animals (6 in SHAM+V, 7 in AMI+V and 8 AMI+3i-1000). For histological analysis, 4 animals were randomly selected from each group. The results are averages ± SEM. Significance (p) was calculated by one-way ANOVA followed by a least significant difference post hoc test. Comparison † SHAM+V vs. AMI+V; ‡ AMI+V vs. AMI+3i-1000. ANP, atrial natriuretic peptide; BNP, B-type natriuretic peptide; HW/BW, heart weight to body weight.

	SHAM+V		AMI+V		†	AMI+3i-1000		‡
	Average	±SEM	Average	±SEM	p	Average	±SEM	p
ANP	1,00	0,20	6,26	1,41	0,001	5,02	1,07	0,361
BNP	1,00	0,18	1,28	0,25	0,426	1,04	0,20	0,462
GATA4	1,00	0,07	0,55	0,11	0,013	0,75	0,14	0,207
NKX2-5	1,00	0,08	0,53	0,13	0,033	0,87	0,18	0,093
Fibrosis	10,33	3,67	20,28	0,53	0,006	18,50	0,28	0,562
Ki67+ cells	1,40	0,93	2,00	0,53	0,487	3,35	0,47	0,132
c-kit+ cells	0,83	0,55	0,78	0,54	0,980	7,78	2,64	0,004
HW/BW (%)	0,34	0,01	0,39	0,02	0,021	0,36	0,01	0,219

Table S5. The echocardiographic parameters of rats that were treated with angiotensin II (33.3 µg/kg/h s.c.) and vehicle or angiotensin II and the compound 3i-1000 (30 mg/kg/day i.p) for two weeks. Echocardiographic measurements were performed at 2 weeks. The number of animals in each group is shown in brackets. The results are averages ± SEM. Significance (p) was calculated by independent samples t-test. IVRT, isovolumic relaxation time; LV, left ventricle; d, diastolic; s, systolic; BPM, beats per minute; IVS, intra ventricular septum; LVPW, left ventricular posterior wall; MeanWT, mean wall thickness.

	Vehicle (6)		3i-1000 (6)		
	Average	±SEM	Average	±SEM	p
E' (cm/s)	95,17	5,84	71,17	2,88	0,004
A' (cm/s)	55,67	2,65	62,67	7,22	0,384
E'/A'	1,74	0,16	1,21	0,14	0,030
IVRT (ms)	28,17	1,40	24,83	1,05	0,086
LV;d (mm)	7,28	0,24	6,28	0,32	0,032
LV;s (mm)	4,23	0,11	3,25	0,28	0,016
Ejection Fraction (%)	77,93	1,72	84,88	1,72	0,017
Fractional Shortening (%)	41,82	1,63	48,95	1,99	0,020
Heart Rate (BPM)	247,17	8,52	295,00	5,86	0,001
LV Mass (mg)	1054,17	88,18	960,00	51,38	0,378
IVS;d (mm)	1,93	0,08	2,13	0,04	0,044
IVS;s (mm)	3,45	0,09	3,50	0,13	0,759
LVPW;d (mm)	1,90	0,12	2,08	0,10	0,260
LVPW;s (mm)	3,15	0,15	3,53	0,15	0,103
MeanWT;d (mm)	1,95	0,07	2,13	0,06	0,062
MeanWT;s (mm)	3,33	0,08	3,55	0,11	0,153

Table S6. The mRNA measurements of rats that were treated with angiotensin II (33.3 µg/kg/h s.c.) and vehicle or angiotensin II and the compound 3i-1000 (30 mg/kg/day i.p) for two weeks. The left ventricular tissues were analysed at the end of the experiment at two weeks. The number of animals in each group is shown in brackets. The results are averages ± SEM. Significance (p) was calculated by independent samples t-test. ANP, atrial natriuretic peptide; BNP, B-type natriuretic peptide; LV mass/BW, left ventricular mass to body weight.

	Vehicle (6)		3i-1000 (6)		
	Average	±SEM	Average	±SEM	p
ANP	1,00	0,48	0,27	0,10	0,163
BNP	1,00	0,20	0,73	0,21	0,377
GATA4	1,00	0,27	0,54	0,11	0,170
NKX2-5	1,00	0,25	0,68	0,20	0,339
Bodyweight (g)	307,67	9,78	295,50	6,63	0,327
LV mass/ BW (mg/g)	3,41	0,23	3,25	0,18	0,607

Table S7. The echocardiographic parameters of rats treated either with vehicle or compound 3i-1000 (30 mg/kg/day i.p.) for one week before ischemia-reperfusion (I/R) or sham-operation (SHAM). Following 30 min of ischemia, the slipknot was released and the myocardium was reperfused for 24 h. Echocardiographic measurements were performed at 24 h. The number of animals in each group is shown in brackets. The results are averages ± SEM. Significance (p) was calculated by one-way ANOVA followed by a least significant difference post hoc test. Comparison SHAM+V vs. I/R+V; ‡ I/R+V vs. I/R+3i-1000. IVRT, isovolumic relaxation time; d, diastolic; s, systolic; BPM, beats per minute; LV, left ventricle; IVS, intra ventricular septum; LVID, left ventricular internal diameter; LVPW, left ventricular posterior wall.

	SHAM+V (4)		I/R+V (15)		†	I/R+3i-1000 (15)		‡
	Average	±SEM	Average	±SEM	p	Average	±SEM	p
E' (mm/s)	-16,20	1,89	-22,19	1,73	0,072	-18,98	1,27	0,134
A' (mm/s)	-15,86	2,04	-18,34	1,74	0,500	-18,83	1,70	0,837
E'/A'	1,04	0,10	1,38	0,19	0,295	1,09	0,10	0,168
IVRT (ms)	17,01	0,90	17,03	0,68	0,994	17,02	0,83	0,997
Diameter;d (mm)	3,85	0,22	4,40	0,05	0,001	4,30	0,06	0,258
Diameter;s (mm)	2,90	0,22	3,52	0,08	0,007	3,37	0,11	0,284
Ejection Fraction (%)	48,89	6,13	40,66	2,76	0,229	43,41	3,35	0,532
Fractional Shortening (%)	24,64	3,76	19,99	1,58	0,238	21,65	1,91	0,512
Heart Rate (BPM)	441,69	25,28	492,35	11,18	0,097	476,06	15,69	0,403
LV Mass (mg)	109,48	10,05	125,20	6,42	0,316	128,28	7,98	0,760
Volume;d (µl)	65,04	8,69	88,01	2,48	0,001	83,24	2,93	0,259
Volume;s (µl)	33,25	6,57	52,30	3,03	0,014	47,52	3,63	0,320
IVS;d (mm)	0,74	0,04	0,72	0,04	0,854	0,68	0,05	0,573
IVS;s (mm)	1,03	0,06	0,92	0,06	0,456	0,92	0,06	0,930
LVID;d (mm)	3,85	0,23	4,32	0,06	0,004	4,25	0,07	0,461
LVID;s (mm)	2,91	0,21	3,46	0,09	0,019	3,32	0,11	0,327
LVPW;d (mm)	0,78	0,05	0,72	0,03	0,287	0,78	0,03	0,124
LVPW;s (mm)	0,96	0,07	0,92	0,04	0,677	1,01	0,05	0,196

Table S8. The mRNA and histological measurements of rats treated either with vehicle or compound 3i-1000 (30 mg/kg/day i.p.) for one week before ischemia-reperfusion (I/R) or sham-operation (SHAM). Following 30 min of ischemia, the slipknot was released and the myocardium was reperfused for 24 h. The left ventricular tissues were analysed at the end of the experiment. The number of animals in each group is shown in brackets. The results are averages ± SEM. Significance (p) was calculated by one-way ANOVA followed by a least significant difference post hoc test. Comparison SHAM+V vs. I/R+V; ‡ I/R+V vs. I/R+3i-1000. ANP, atrial natriuretic peptide; BNP, B-type natriuretic peptide; COL1A1, collagen 1A1.

	SHAM+V (4)		I/R+V (15)		†	I/R+3i-1000 (15)		‡
	Average	±SEM	Average	±SEM	p	Average	±SEM	p
ANP	1,00	0,40	1,81	0,21	0,041	1,27	0,12	0,039
BNP	1,00	0,23	1,31	0,13	0,257	1,09	0,12	0,225
GATA4	1,00	0,20	0,64	0,05	0,004	0,59	0,04	0,547
NKX2-5	1,00	0,16	0,63	0,05	0,003	0,62	0,04	0,865
COL1A1	1,00	0,14	1,21	0,13	0,436	1,11	0,13	0,581
Apoptosis (tunel)	0,90	0,24	1,91	0,21	0,052	1,77	0,27	0,684

Table S9. The echocardiographic parameters of rats that underwent acute myocardial infarction (AMI) or sham-operation (SHAM) and were treated either with vehicle or compound 3i-1000 loaded TOPSi 7µm particles. Echocardiographic measurements were performed at one week. The number in each group is shown in brackets. The results are averages ± SEM. Significance (p) was calculated by one-way ANOVA followed by a least significant difference post hoc test. Comparison † SHAM+TOPSi vs. AMI+TOPSi; ‡ AMI+TOPSi vs. AMI+3i-1000-TOPSi; ¥ SHAM+3i-1000-TOPSi vs. AMI+3i-1000-TOPSi. IVRT, isovolumic relaxation time; d, diastolic; s, systolic; BPM, beats per minute; LV, left ventricle; IVS, intra ventricular septum; LVID, left ventricular internal diameter; LVPW, left ventricular posterior wall.

	SHAM+TOPSi (7)		SHAM+3i-1000-TOPSi (7)		¥	AMI+TOPSi (6)		†	AMI+3i-1000-TOPSi (7)		‡
	Average	±SEM	Average	±SEM	p	Average	±SEM	p	Average	±SEM	p
E' (mm/s)	-39,52	1,11	-38,48	3,14	0,054	-31,34	3,31	0,057	-30,53	3,25	0,844
A' (mm/s)	-18,60	2,33	-34,42	5,32	0,006	-18,49	1,82	0,984	-19,22	3,54	0,890
E'/A'	2,37	0,35	1,30	0,25	0,179	1,74	0,16	0,119	1,82	0,27	0,838
IVRT (ms)	23,64	0,83	23,15	1,33	0,752	33,67	10,33	0,154	25,24	1,76	0,228
Diameter;d (mm)	8,11	0,19	8,29	0,15	0,249	9,30	0,33	0,046	8,93	0,65	0,515
Diameter;s (mm)	5,64	0,21	5,56	0,20	0,009	7,63	0,59	0,005	7,31	0,62	0,617
Ejection Fraction (%)	55,97	2,44	59,30	2,44	0,002	35,23	7,61	0,008	35,08	5,95	0,984
Fractional Shortening (%)	30,57	1,68	32,99	1,85	0,002	18,47	4,56	0,011	18,13	3,51	0,938
Heart Rate (BPM)	267,74	8,73	278,97	9,13	0,526	264,30	14,20	0,840	289,41	14,10	0,150
LV Mass (mg)	978,99	46,74	928,47	46,98	0,892	819,46	97,25	0,088	916,64	55,52	0,290
Volume;d (µl)	356,58	18,43	373,61	15,22	0,170	485,03	37,23	0,048	457,27	71,67	0,656
Volume;s (µl)	157,82	13,12	152,70	12,24	0,008	321,92	47,64	0,004	297,02	53,23	0,636
IVS;d (mm)	1,54	0,18	1,45	0,08	0,113	0,84	0,18	0,007	1,11	0,19	0,261
IVS;s (mm)	2,13	0,22	2,23	0,08	0,029	1,18	0,29	0,007	1,51	0,26	0,312
LVID;d (mm)	8,03	0,21	8,05	0,15	0,140	9,25	0,36	0,044	8,89	0,65	0,531
LVID;s (mm)	5,59	0,23	5,40	0,19	0,006	7,62	0,61	0,004	7,28	0,61	0,604
LVPW;d (mm)	1,59	0,07	1,49	0,07	0,823	1,35	0,17	0,141	1,46	0,11	0,490
LVPW;s (mm)	2,30	0,10	2,35	0,10	0,085	1,77	0,27	0,049	1,91	0,22	0,593

Table S10. The mRNA and histological measurements of rats that underwent acute myocardial infarction (AMI) or sham-operation (SHAM) and were treated either with vehicle or compound 3i-1000 loaded TOPSi 7 µm particles. The left ventricular tissues were analysed at the end of the experiment at one week. The number of animals in each group is shown in brackets. The results are averages ± SEM. Significance (p) was calculated by one-way ANOVA followed by a least significant difference post hoc test. Comparison † SHAM+TOPSi vs. AMI+TOPSi; ‡ AMI+TOPSi vs. AMI+3i-1000-TOPSi; ¥ SHAM+3i-1000-TOPSi vs. AMI+3i-1000-TOPSi. ANP, atrial natriuretic peptide; BNP, B-type natriuretic peptide; COL1A1, collagen 1A1; OPN, osteopontin; HW/BW, heart weight to body weight.

	SHAM+TOPSi (7)		SHAM+3i-1000-TOPSi (7)		¥	AMI+TOPSi (6)		†	AMI+3i-1000-TOPSi (7)		‡
	Average	±SEM	Average	±SEM	p	Average	±SEM	p	Average	±SEM	p
ANP	1,00	0,12	0,64	0,11	0,006	4,32	1,20	0,001	3,08	0,46	0,157
BNP	1,00	0,11	1,02	0,23	0,016	0,52	0,11	0,030	0,50	0,05	0,921
GATA4	1,00	0,16	1,32	0,27	0,024	0,93	0,13	0,789	0,72	0,07	0,420
NKX2-5	1,00	0,25	0,43	0,15	0,603	0,30	0,10	0,007	0,31	0,11	0,953
COL1A1	1,00	0,23	0,38	0,08	0,021	5,06	1,09	0,000	2,46	0,68	0,007
TNF-alpha	1,00	0,42	1,04	0,40	0,674	1,75	0,58	0,295	1,32	0,54	0,549
OPN	1,00	0,23	0,29	0,07	0,070	11,16	4,26	0,023	7,89	4,21	0,442
HW/BW	3,75	0,14	3,71	0,04	0,006	4,37	0,27	0,036	4,54	0,26	0,569

Table S11. The mRNA measurements of the left ventricular endocardial layer of rats in response to isoprenaline-induced myocardial ischemia after treatment with 3i-1000 loaded nanoparticles. The isoprenaline was injected s.c. 24 h before i.v administration of the control particles or 3i-1000 loaded particles. The samples were collected 4 h after particle injections. The number of animals in each group is shown in brackets. The results are averages ± SEM. Significance (p) was calculated by independent samples t-test. ANP, atrial natriuretic peptide; BNP, B-type natriuretic peptide; COL1A1, collagen 1A1; IL6, interleukin-6; OPN, osteopontin; TGFB1, transforming growth factor beta 1; TNF-alpha, tumour necrosis factor alpha.

	Control (4-5)		3i-1000 (4-5)		†
	Average	±SEM	Average	±SEM	p
ANP	1,00	0,02	0,92	0,01	0,010
BNP	1,00	0,07	0,75	0,07	0,044
GATA4	1,00	0,00	1,08	0,06	0,236
NKX2-5	1,00	0,05	1,05	0,09	0,673
COL1A1	1,00	0,00	0,75	0,06	0,013
IL6	1,00	0,02	0,50	0,04	0,000
OPN	1,00	0,16	0,55	0,06	0,024
TGFB1	1,00	0,15	0,88	0,12	0,525
TNF-alpha	1,00	0,08	0,86	0,05	0,150

Table S12. Taqman probes used on RT-PCR. m, mouse; r, rat; ANP, atrial natriuretic peptide; BNP, B-type natriuretic peptide; COL1A1, collagen 1A1; TNF-alpha, tumour necrosis factor alpha; OPN, osteopontin.

Messinger RNA	Probe	Sequence
mANP	Forward	GAAAAGCAAACTGAGGGCTCTG
	Reverse	CCTACCCCCGAAGCAGCT
	Fluorogenic probe	TCGCTGGCCCTCGGAGCCT
rANP	Forward	GAAAAGCAAACTGAGGGCTCTG
	Reverse	CCTACCCCCGAAGCAGCT
	Fluorogenic probe	TCGCTGGCCCTCGGAGCCT
mBNP	Forward	AGGCGAGACAAGGGAGAACA
	Reverse	GGAGATCCATGCCGCAGA
	Fluorogenic probe	CATCATTGCCTGGCCCATCGC
rBNP	Forward	TGGGCAGAAGATAGACCGGA
	Reverse	ACAACCTCAGCCCGTCACAG
	Fluorogenic probe	CGGCGCAGTCAGTCGCTTGG
m,rGATA4	Forward	AAGGCTATGCATCTCCTGTCACT
	Reverse	CCAGGCTGTTCCAAGAGTCC
	Fluorogenic probe	ACATCGCAGGCCAGCTCCAAGC
mNKX2-5	Forward	CCTCGGGCGGATAAAAAAGA
	Reverse	CGGCTTTGTCCAGCTCCA
	Fluorogenic probe	CTGTGCGCGCTGCAGAAGGC
rNKX2-5	Forward	CCTCGGGCGGATAAGAAAGA
	Reverse	CGGCTTTGTCCAGCTCCA
	Fluorogenic probe	CTGTGCGCGCTGCAGAAGGC
m,r18 S	Forward	TGGTTGCAAAGCTGAAACTTAAAG
	Reverse	AGTCAAATTAAGCCGCAGGC
	Fluorogenic probe	CCTGGTGGTGCCCTTCCGTCA
mCOL1A1	Forward	CCCTGGCCTTGGAGGAA
	Reverse	CACGGAAACTCCAGCTGATTTT
	Fluorogenic probe	CTTTGCTTCCCAGATGTCCTATGGCTATGATG
rCOL1A1	Forward	CCCCTTGGTCTTGGAGGAA
	Reverse	GCACGGAAACTCCAGCTGAT
	Fluorogenic probe	CTTTGCTTCCCAGATGTCCTATGGCTATGATG
rTNF-alpha	Forward	GACAAGGCTGCCCCGACTA
	Reverse	CTCCTGGTATGAAGTGGCAAATC
	Fluorogenic probe	TGCTCCTCACCCACACCGTCAGC
rOPN	Forward	AATCGCCCCCACAGTCG
	Reverse	CCTCAGTCCGTAAGCCAAGC
	Fluorogenic probe	TGTCCCTGACGGCCGAGGTGA
